# Supplement Based on Fermented Milk Permeate for Feeding Newborn Calves: Influence on Blood, Growth Performance, and Faecal Parameters, including Microbiota, Volatile Compounds, and Fatty and Organic Acid Profiles

**DOI:** 10.3390/ani11092544

**Published:** 2021-08-30

**Authors:** Laurynas Vadopalas, Egle Zokaityte, Paulina Zavistanaviciute, Romas Gruzauskas, Vytaute Starkute, Ernestas Mockus, Jolita Klementaviciute, Modestas Ruzauskas, Vita Lele, Darius Cernauskas, Dovile Klupsaite, Agila Dauksiene, Antanas Sederevicius, Sarunas Badaras, Elena Bartkiene

**Affiliations:** 1 Faculty of Animal Sciences, Institute of Animal Rearing Technologies, Lithuanian University of Health Sciences, Mickeviciaus Str. 9, LT-44307 Kaunas, Lithuania; laurynas.vadopalas@lsmu.lt (L.V.); egle.zokaityte@lsmuni.lt (E.Z.); paulina.zavistanaviciute@lsmuni.lt (P.Z.); vytaute.starkute@lsmuni.lt (V.S.); ernestas.mockus@lsmuni.lt (E.M.); jolita.klementaviciute@lsmuni.lt (J.K.); vita.lele@lsmuni.lt (V.L.); darius.cernauskas@lsmuni.lt (D.C.); dovile.klupsaite@lsmuni.lt (D.K.); agila.dauksiene@lsmuni.lt (A.D.); sarunas.badaras@lsmuni.lt (S.B.); 2 Department of Food Safety and Quality, Faculty of Veterinary Medicine, Lithuanian University of Health Sciences, Mickeviciaus Str. 9, LT-44307 Kaunas, Lithuania; 3 Department of Food Science and Technology, Kaunas University of Technology, Radvilenu Rd. 19, LT-50254 Kaunas, Lithuania; romas.gruzauskas@ktu.lt; 4 Faculty of Veterinary, Institute of Microbiology and Virology, Lithuanian University of Health Sciences, Mickeviciaus Str. 9, LT-44307 Kaunas, Lithuania; modestas.ruzauskas@lsmuni.lt; 5 Department of Anatomy and Physiology, Faculty of Veterinary, Lithuanian University of Health Sciences, Mickeviciaus Str. 9, LT-44307 Kaunas, Lithuania; antanas.sederevicius@lsmuni.lt

**Keywords:** calves, fermented milk permeate, faeces, blood parameters, short-chain fatty acids, volatile compounds

## Abstract

**Simple Summary:**

Gastrointestinal infections and diarrhoea are the main health issues in young calves. The application of microbial products containing probiotics and prebiotics may lead to better management of the gut microbiome and improved calf health. After fermentation with selected lactic acid bacterial strains, milk permeate (a dairy industry by-product) contains lactic acid bacteria and prebiotics, both of which possess viable antimicrobial properties. We hypothesised that fermented milk permeate could be a prospective feed supplement for newborn calves. A 14-day experiment was conducted in which a group of newborn calves were given a supplement of milk permeate fermented with *Lactobacillus uvarum* LUHS245. A significantly higher count of lactic acid bacteria, a lower total count of enterobacteria, a higher species variety, and greater concentrations of both propionic acid and dry matter were found in the faeces of the calves fed with fermented milk permeate compared with a control group. Most of the fatty acids and volatile compounds in the faeces differed significantly between the two groups. The results suggest that supplementing the calves’ feed with fermented milk permeate has a positive effect on certain health parameters but no influence on blood parameters and growth performance.

**Abstract:**

The aim of this study was to assess the effect of a feed supplement, namely milk permeate (MP) fermented with *Lactobacillus uvarum* LUHS245, on the newborn calves’ growth performance and blood and faecal parameters, including microbiota and volatile compound and fatty acid profiles. Ten female Holstein calves in the control group (CON group) were fed with a standard milk replacer diet and colostrum only, from day 2 to 14 of life, while 10 calves of the treated group (MP group) were fed with the same diet supplemented with 50 mL of the fermented MP. After 14 days, there were no significant differences between the groups in blood parameters, growth performance, or faecal pH. There was a significantly higher percentage of live lactic acid bacteria (by 17.02%), a lower percentage of enterobacteria (by 10.38%), a higher overall number of probiotic bacteria, a 1.7-fold higher species variety, and a higher content of dry matter in the faeces of the MP group (*p* < 0.05). The fatty acid and volatile compound profiles differed significantly between the groups. The results suggest that supplementing calves’ feed with fermented milk permeate has a positive effect on certain health parameters but not on blood parameters or growth performance.

## 1. Introduction

Global climate change influences people, ecosystems, and livelihoods across the world. However, the philosophy of sustainability also espouses broader principles that support the just treatment of farm workers and food pricing to provide the farmer with a liveable income [1]. To ensure these principles, the effective valorisation of food industry by-products has become very important.

Our previous studies have shown that dairy industry by-product milk permeate (MP) could be effectively valorised to antimicrobial functional properties possessing ingredients for humans and animal nutrition [2]. MP is usually obtained after removing milk protein and fat by an ultrafiltration process [3]. This by-product contains unmodified lactose, minerals, and serum proteins. However, fermentation with selected lactic acid bacteria (LAB) strains can improve the physicochemical, antimicrobial, functional, and sensory characteristics of MP [4]. During the fermentation process, lactose in MP is converted to functional compounds, including galactooligosaccharides (GOSs) and high concentrations of viable LAB. This process results in animal feed with GOS-enriched antimicrobial properties [2,5]. For this reason, we hypothesised that fermented MP is a prospective feed supplement for newborn calves.

Probiotics are widely used in animal feed supplements [6] for a variety of purposes, one of which is their ability to protect against infections caused by pathogenic bacteria [7,8]. Because probiotics promote the establishment of a beneficial gut flora and inhibit the growth of pathogenic bacteria in the intestine, they are expected to improve livestock health [9,10]. Probiotics for livestock have been studied using many different LAB strains individually and in combination [11,12,13]. In this study, we focused on LAB strains with antimicrobial properties used for MP, with a view to probiotics potentially reducing or replacing the use of antibiotics in animal husbandry [14]. Diarrhoea is one of the most common health problems in young dairy calves [15], and the use of probiotics has been proposed as an alternative for the prevention and alleviation of intestinal disorders, as well as immunomodulators [16]. Another promising approach is the use of prebiotics in the production of healthier feeds [17]. Prebiotics are nondigestible compounds that have a positive effect on the host by promoting the growth and activity of one or more bacteria in the colon [18]. Nondigestible oligosaccharides (NDOs) are the most promising prebiotics; this group include GOSs, which could be used in animal production as a potential alternative to antimicrobial growth promoters [19]. They participate in the formation of a healthier microbiota in which lactobacilli predominate and can exert health-promoting effects at the expense of more harmful organisms [20]. Finally, probiotic and prebiotic supplements have the greatest impact in the first weeks or months of a calf’s life [16,21]. This may lead to the instability of their microbial populations, while the microbiota later in life is more stable and difficult to affect [22]. Because the gut microbiome is a key factor affecting gut health [23], the use of microbial products early in life could become a preferential treatment for improving calf health [24]. For this study, we conducted a 14-day experiment with newborn calves. An experimental group was given fermented MP containing both prebiotics (GOSs) and an LAB strain with viable antimicrobial properties.

Although at the present time there is much information available on the digestion and absorption processes of the major macro- and micronutrients [25], there is still a wide gap in the literature when it comes to biomarkers for indicating the health status of young animals. Biomarkers could show how well the gastrointestinal tract performs its essential digestive and absorptive functions [26]. Although digestion and absorption are regularly assessed in research contexts, many of the technologies and equipment used for this purpose are not available as a point-of-care tool for providing real-time assessment on the farm. However, testing for faecal biomarkers, such as volatile compounds (VCs), can be useful in determining the efficiency of the digestion and absorption processes [27,28,29].

The aim of this study was to evaluate the influence of a MP supplement fermented with *Lactobacillus uvarum* LUHS245 on the newborn calves’ growth performance, blood, and faecal parameters, including microbiota and the VC and fatty acid (FA) profiles.

## 2. Materials and Methods

### 2.1. Characteristics of the Fermented Milk Permeate

MP was obtained from the ‘Pienas LT’ agricultural cooperative (Biruliskes, Kaunas district, Lithuania) and stored at −18 °C until use. Our previous studies showed that *L. uvarum* LUHS245 could be used in dairy farms for improvement of animals’ health [30]. MP is able to act as a substrate for sustainable multiplication of the LUHS245 bacilli. During the MP fermentation, the LUHS245 strain converts MP lactose to GOS, resulting in additional antimicrobial characteristics of the MP [2]. The method for preparing fermented MP is given in Zokaityte et al. [2]. Parameters of the biotreated MP used in this study for feeding newborn calves are given in Tables S1–S3 (Supplementary File 1).

### 2.2. Feeding Experiment

A total of 20 female Holstein calves were randomly allocated to two equal groups on the day of birth (day 1). All the calves received the first colostrum from their dams during day 1 and were enrolled in the study on day 2. Calves of the control (CON) group were fed only with colostrum and a standard milk replacer (22.5% crude protein, 18% fat, 9% ash, 1.75% lysine, 0.55% methionine, and 0.5% cysteine on a dry matter basis). Calves of the MP group were fed with the same diet supplemented with 50 mL of the fermented MP. For the MP group, the fermented MP was mixed with the milk replacer (130 g/L reconstituted in hot water at 65 °C).

Each calf was placed in an individual outdoor box (2.00 m × 1.25 m), with free access to warm water. Calves were fed individually once a day, at 7:00 a.m., for 14 days. They were fed from a bucket with 8–10 l of unmedicated milk replacer at 39 °C—either with or without the fermented MP.

### 2.3. Blood Analysis

Calves were bled (5 mL) aseptically from the jugular vein into vacuum blood tubes (BD Vacutainer^®^, Weymouth, UK) on days 2 and 14 of the experiment before the morning feeding. The samples taken on day 2 were before the feeding experiment started and were used for baseline measurements. The tubes with clot activator were used for biochemical examination of the blood. The parameters assessed included lactate concentration and aspartate aminotransferase (AST). These parameters were quantified using an automatic biochemical analyser in an accredited laboratory (Kaunas, Lithuania).

### 2.4. Microbiological Analysis of Faecal Samples

Animals’ faeces were collected on days 2 and 14 (kept at +4 °C with a medium (Faecal Enteric Plus, Oxoid, Basingstoke, UK)), and analysed on the same day. Evaluation of total count of aerobic and facultative anaerobic microorganisms (TCM), LAB, TBC, total count of enterobacteria [TCE], and yeast/mould [Y/M] counts was performed according to the methods described by Zavistanaviciute et al. [30].

### 2.5. Metagenomic Analysis of Faecal Samples

Faecal samples for microbial profiling were taken before and at the end of the experiment. The Quick-DNA Faecal/Soil Microbe Kit (Zymo Research, Irvine, CA, USA) was used according to the manufacturer’s instructions for total DNA extraction. The initial quantity and quality of the DNA was controlled using the Nano Drop 2000 (Thermo Fisher, Waltham, MA, USA) spectrophotometer. The DNA from each calf was pooled in equal proportions into two separate samples representing the CON and MP groups. Metagenomic analysis was performed in an independent service laboratory (Baseclear, Leiden, The Netherlands). The results of the taxonomic classification were presented on the interactive online platform.

### 2.6. Evaluation of the Faecal pH, Dry Matter, and Colour Coordinates

The pH of samples was analysed with a pH meter (Inolab 3, Hanna Instruments, Italy). The dry matter (DM) of the samples was determined after drying the faeces at 103 ± 2 °C to a constant weight. The colour coordinates were fixed at three different points of the sample surface using the CIE L*a*b* system (CromaMeter CR-400, Conica Minolta, Tokyo, Japan).

### 2.7. Analysis of the Faecal Volatile Compounds by Gas Chromatography–Mass Spectrometry

Faecal samples for gas chromatography (GC) analysis were prepared by using solid phase microextraction (SPME). A solid phase microextraction device with Stableflex (TM) fibre, coated with a 50-µm DVB-PDMS-Carboxen™ layer (Supelco, Bellefonte, PA, USA), was used for sample preparation. For headspace extraction, 1 g of sample in the 20-mL extraction vial, sealed with a polytetrafluoroethylene septum, was thermostated at 60 °C for 15 min, thereby exposing the fibre in the headspace. The fibre was then exposed to the headspace of the vial for a further 10 min. The desorption time was 2 min.

For gas chromatography–mass spectrometry (GC–MS), a GCMS-QP2010 (Shimadzu, Japan) was used. The gas chromatograph was equipped with an AOC-5000 Plus Shimadzu autosampler, upgraded with an SPME analysis kit. The following analysis parameters were used: for the ionisation of analytes at 70 eV; for separation of VCs, a low-polarity Rxi^®^-5MS column (Restek, Bellefonte, PA, USA); injector temperature 250 °C, ion source, and interface temperatures 220 °C and 280 °C, respectively; the temperature gradient: from 35 °C (5 min hold) to 200 °C (10 °C/min) and then up to 280 °C (25 °C/min) (5 min hold); carrier gas was helium (99.999% detector purity, Linde, Vilnius, Lithuania), pressure of 6.61 psi (45.6 kPa); column flow 0.97 mL/min; the compounds were identified according to the mass spectra libraries (NIST11, NIST11S, FFNSC2).

### 2.8. Fatty Acid Profile Analysis

The FA composition of the faecal samples was determined using gas chromatography–flame ionisation detection (GC–FID; Agilent 6890N Gas Chromatograph, Agilent Technologies, Santa Clara, CA, USA). First, FA were dissolved in cyclohexane (100 mg in 4 mL), then methyl esters of FA were prepared by transmethylation using 8 mL of 1.5% sulphuric acid in methanol, and kept at 60 °C for 12 h. The samples were cooled, shaken for 30 s, and then centrifuged for 10 min at 3000× *g* at 17 °C. They were then injected with 100 µL of the upper portion of the supernatant (previously diluted with cyclohexane in the ratio of 1:9) into a capillary BPX90 column (60 m × 0.32 mm ID × 0.25-µm film thickness; SGE, Santa Clara, CA, USA). The following parameters were used: flame ionisation detector: 280 °C; H_2_ flow: 40 mL/min; air flow: 450 mL/min; helium (carrier gas) flow: 1 mL/min; injector: 250 °C (split 1:10); oven temperature: 50 °C for 2 min, then 4 °C/min to 245 °C, and then 245 °C for 15 min. Each FA was identified according to its retention time and is expressed as a percentage of the total peak area of all FA in the sample.

### 2.9. The Evaluation of Short-Chain Fatty Acids in the Faeces of Newborn Calves

Short-chain fatty acids (SCFA) were determined as described by Zhao et al. [31] with some modifications. The sample (1 g) in 10 mL of water was homogenised for about 3 min. The resulting solution was adjusted to a pH of 2–3 with 5 M HCl and then kept at room temperature for 10 min with occasional shaking. The solution was then centrifuged for 20 min at 3000× *g*, yielding a clear supernatant. One microlitre of the supernatant was injected into the GC-2010 Plus combined with the GCMS-QP2010 for analysis.

### 2.10. Evaluation of the Calves’ Growth Performance

Each calf’s body weight (BW) was recorded at 2 and 14 days of age, using an electronic weighing scales (model BF/E 1425E, Technosystem, Via Toscana, Certaldo FI, Italy).

### 2.11. In Vivo Experiment Ethical Guidelines

The calves were housed indoors and were individually tethered and cared for in accordance with the Lithuanian State Food and Veterinary Service Requirements. Research was carried out in accordance with both the Republic of Lithuania Act (6 November 1997) regulating animal care and maintenance and its subsequent legal amendment (Act 8-500) [32].

### 2.12. Statistical Analysis

All analytical analysis of animals’ samples were performed in triplicate. SPSS package (Version 15.0, SPSS, Chicago, IL, USA) was used for statistical analysis. Differences between the most prevalent bacterial genera among the two groups of calves were assessed using the Z-Test Calculator for Two Population Proportions (Social Science Statistics). All results were considered statistically significant at *p* ≤ 0.05.

## 3. Results and Discussion

### 3.1. Blood Parameters and Growth Performance of the Newborn Calves

The blood parameters and growth performance of newborn calves fed with milk replacer only (CON group) or supplemented with fermented MP (MP group) are shown in Table 1. There were no significant differences between the CON and MP groups in blood parameters at the beginning or at the end of the experiment. These results are in agreement with the results of Dar et al. [7], who found no major differences in serum AST activity between the control, prebiotic, and probiotic dietary treatment groups at various points during an experiment on the feeding of calves. Similarly, Takagi et al. [33] found no substantial difference between probiotic and control groups of calves. Some of the probiotic LAB strains have been shown to produce only L-lactate [34]. Formation of D-lactate may have implications in calf diarrhoea because D-lactic acidosis is often present [35], and D-lactate is poorly metabolised by mammals [36].

After 14 days of the present experiment, the CON14 and MP14 groups had gained weight by 15.1% and 15.7%, respectively, with no significant difference between the two groups. In our study, MP contained GOSs and viable counts of *L. uvarum* LUHS245. In other studies, researchers have shown that probiotics could increase the growth of ruminants, while the growth performance of calves could also be improved by the use of prebiotics, such as mannan oligosaccharides, fructooligosaccharides, and galactosyl-lactose [6,21]. However, previous studies of the impact of probiotics and prebiotics on the growth of calves have produced inconsistent results. The review of Frizzo et al. [37] revealed that probiotic LAB improved body-weight gain in young calves. By contrast, other researchers have shown that the use of *Lactobacillus acidophilus*, *Lactobacillus plantarum*, or *Lactobacillus lactis* did not influence calf growth [6]. Heinrichs et al. [38] showed that calf health and growth measurements were not influenced by prebiotic treatment because the calves in that study were all generally healthy. Uyeno et al. [21] reported that prebiotics may not elicit any detectable change in body weight or other health measures when calves are primarily healthy.

### 3.2. Microbiological Parameters of the Newborn Calves’ Faecal Samples

The microbiological parameters of the faecal samples from the calves are shown in Table 2. Comparison of faeces from the CON and MP groups at the beginning of the experiment revealed no significant differences between the two groups in the LAB count, the TCM, the *Enterococcus faecalis* count, or the Y/F count. However, after 14 days of treatment, the faeces from the MP group showed a 17.02% higher LAB count.

It has been reported that calves treated with probiotics had lower faecal counts of *Clostridium* spp., elevated counts of enterococci, and inconclusive changes in the counts of *Faecalibacterium*, *Bifidobacterium*, and *Bacillus* spp. [39]. Dar et al. [7] reported that both a probiotic (*L. acidophilus*) and a prebiotic (mannan oligosaccharide) reduced faecal coliform and *E. coli* counts in calves. According to Heinrichs et al. [38], the concentrations of beneficial bacteria in faeces were unaffected by probiotic treatment, although prebiotic-fed calves had higher populations of beneficial bacteria. The prebiotic feed additive was found to have a positive effect on the microbial landscape of faeces in calves, with increased lactobacilli and bifidobacterial counts as well as decreases in *Escherichia* [40]. To evaluate the influence of the fermented MP supplement on the populations of beneficial bacteria in the newborn calves’ faeces, a metagenomic profile of the faeces was generated during the second phase of the experiment.

### 3.3. Microbial Profiles in the Faeces of Calves

**Figure 1:**
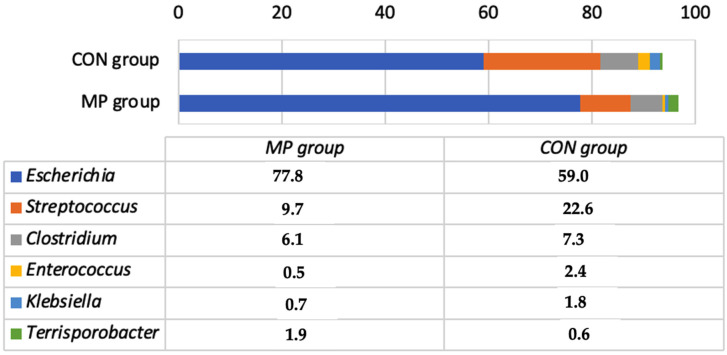
Bacterial genera percentage prevalence in the faeces of calves before the experiment. The genera are included in the list only if the prevalence was at least 1% of the total bacterial reads in the faeces of both groups combined. CON, calves fed with milk replacer only; MP, calves fed with milk replacer and supplemented with fermented milk permeate.

In total, 39,676 and 41,469 bacterial reads were obtained from the CON and MP groups, respectively, before the experiment began. The bacterial composition was similar in both groups of newborn calves (Figure 1). Six genera (*Escherichia*, *Streptococcus*, *Clostridium*, *Enterococcus*, *Klebsiella*, and *Terrisporobacter*) represented >93% of the total bacterial reads.

**Figure 2:**
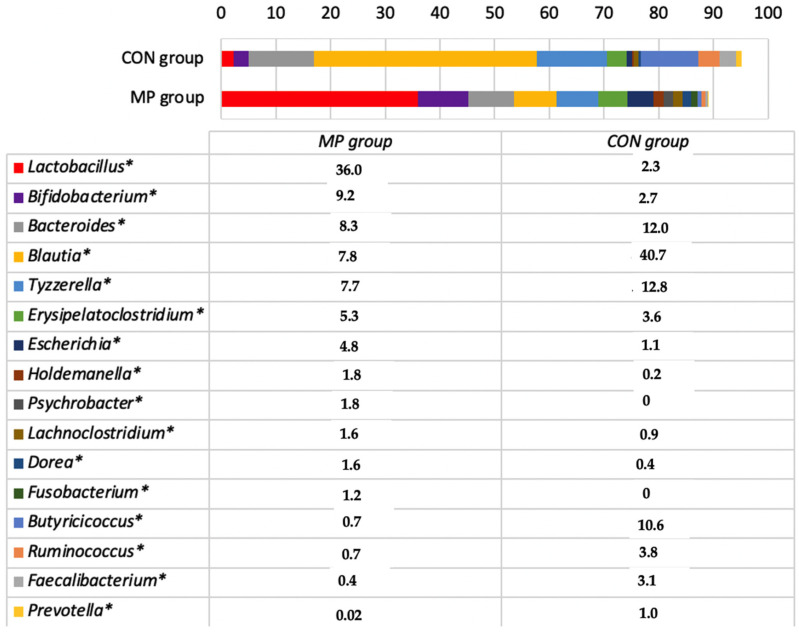
Bacterial genera percentage prevalence in the faeces of calves after the experiment. The genera are included in the list only if the prevalence was at least 1% of the total bacterial reads in the faeces of both groups. CON, calves fed with milk replacer only; MP, calves fed with milk replacer and supplemented with fermented milk permeate.* Significant differences between the groups.

At the end of the experiment, 28,265 and 26,968 bacterial reads were obtained from the faeces of the MP and CON groups, respectively. The microbial profiles differed significantly between the groups (Figure 2).

The most prevalent bacterial genera in the MP group were *Lactobacillus* and *Bifidobacteria*, whereas in the CON group the most prevalent were *Blautia* and *Tyzzerella* (Figure 2). Bacteria of these genera accounted for 45.2% and 53.5% of all bacterial counts in the MP and CON groups, respectively. The other most prevalent genera included *Bacteroides*, *Erysipelatoclostridium*, *Escherichia*, *Butyricicoccus*, *Ruminococcus*, and *Faecalibacterium* (Figure 1, Tables S4 and S5). The number of species with a prevalence of at least 0.01% of the total bacterial reads differed significantly among the groups; 234 species were detected in the MP group and only 138 species in the CON group. The most prevalent species in the MP group were probiotic, including *Lactobacillus amylovorus*, *Bifidobacterium longum*, *Lactobacillus johnsonii*, and *Tyzzerella nexilis*. The most prevalent species in the CON group were *Ruminococcus torques*, *Blautia wexlerae*, *Butyricicoccus pullicaecorum*, and *T. nexilis*. All the bacterial species found in both groups are presented in Tables S6 and S7 (Supplementary File 2). The study therefore demonstrates that the MP supplement has a strong influence on the microbial populations in the gut of calves, resulting in an increase in the numbers of *Lactobacillus* and *Bifidobacterium*. Multiple studies have substantiated the positive influence of these bacteria on animal and human health [41,42,43,44]. However, data on the most prevalent bacterial species detected in calves of control groups are scarce. It is known that *R. torques* may affect intestinal health and contribute to the observed disruption of intestinal barrier functions [41]. There is little or no information regarding the roles of *B. wexlerae* and *Tyzzerella* in calves, whereas *Butyricicoccus* is known to have a positive influence on the health status of calves [45].

### 3.4. Faecal pH, Dry Matter, Texture, and Colour Coordinates

The pH, dry matter, texture, and colour coordinates of the calves’ faeces are given in Table 3. Both groups had a significantly higher pH after 14 days of feeding (by 17.4% and 13.7% in the CON and MP groups, respectively) compared with the beginning of the experiment (*p* = 0.0001). However, the difference in pH between the CON and MP groups was not significant. Fujisawa et al. [46] also reported that addition of prebiotics in feed did not impact the pH of calves’ faeces. After 14 days of feeding, the dry matter content in faeces decreased in both groups. However, there was a significantly higher (*p* = 0.0001) content of dry matter in the MP group compared with the CON group at the end of the experiment. There was a significant increase (*p* = 0.0001) in the L* coordinate of the faeces in both groups after 14 days of treatment, but there was no difference between the groups. The a* coordinate was significantly reduced after 14 days only in the CON group. There were no significant differences between groups or time intervals in the texture or in the b* coordinate of the faeces. In general, the dry matter content was the only parameter that differed between the CON and MP groups. Stefańska et al. [10] demonstrated lower faecal scores for dry matter content and reduced occurrence of diarrhoea in calves fed with probiotics, while Signorini et al. [47] reported more liquid faecal consistency in animals fed with probiotics.

### 3.5. Volatile Compounds in the Calves’ Faeces

The comprehensive faecal VC profile of calves in the CON and MP groups is given in Table S8 (Supplementary File S8). However, due to the large amount of data, only the most prevalent VCs are summarised here.

The following baseline measurements (0) of VCs in faeces in the CON0 group were found in significant concentrations (Table 4), with the following in decreasing amounts: nonanoic acid, 2-nonanone, butanoic acid, acetoin, octanoic acid, and indole. In the MP0 group, the predominant VCs were butanoic acid, indole, nonanoic acid, 2-propyl-1-pentanol, and octanoic acid/2-nonanone (Table 4). The concentration of each VC differed significantly between the groups (*p* < 0.05), with the exception of *n*-decanoic acid. After 14 days of feeding, *p*-cresol, butanoic acid, and indole were the predominant compounds in both groups. The concentration of *p*-cresol was significantly higher (by 20.8%) in the MP14 group compared with the CON14 group (*p* = 0.005). Compared with the beginning of the experiment, there was a 3.4-fold increase in butanoic acid in the CON14 group, while this VC increased only by 12.7% in the MP14 group (*p* = 0.003). There was not a significant difference between the MP14 and CON14 groups in the concentration of indole. However, there were significant differences between the MP14 and CON14 groups in the concentration of all the other VCs (*p* < 0.05), with the exceptions of hexanoic acid, 2-nonanone, and nonanoic acid (Table 4). Moreover, after 14 days of feeding, acetic acid in the faeces decreased by 39.6% and 67.2% in the CON14 and MP14 groups, respectively, whereas acetoin, 2-heptanone, and 2-propyl-1-pentanol were not detected in either group after 14 days.

The production of faecal VCs (volatile fatty acids (VFA), alcohols, and phenolic and aromatic compounds) results from the colonic fermentation of non-utilised nutrients in the small intestine [48]. *p*-Cresol is formed during tyrosine fermentation, while indole is the principal end-product of tryptophan catabolism [49]. VFA maintain gut epithelium functionality and are derived mostly from lactose, non-utilised fibrous nutrients, and starch [50]. Shimomura and Sato [48] stated that the reduced concentration of VFA in the faeces of newborn calves is related to rapid lactic fermentation in the acidic lumen, while Ohya [51] reported increased concentrations of these acids, especially acetic acid, as well as reduction in *E. coli* in the faeces following administration of probiotic bacteria. Higher concentration of VFA in faeces is related to better adaptation of probiotics in the gut of calves and improved utilisation of prebiotics by beneficial gut bacteria [52]. In our study, the total concentration of VFA in the faeces was lower in the MP14 group (43%) compared with the CON14 group (54%). However, there was a significantly higher concentration of acetic acid in the CON14 group (*p* = 0.002).

### 3.6. Fatty Acid Profiles of the Calves’ Faeces

The FA profiles of the calves’ faeces before the experiment and after 14 days of feeding with fermented MP are given in Table 5. There were significant differences between the CON0 and MP0 groups in most of the faecal FA content (*p* < 0.05), with the exceptions of C14:1, C17:1, C18:2, C20:1, and C20:3. The predominant faecal FA were the same in both the CON0 and MP0 groups: C14:0, C16:0, C18:1, C18:2, and C18:3α. After 14 days, C16:0, C18:0, and C18:1 were the predominant faecal FA in the CON14 group, while C16:0, C18:1, C18:2, and C18:3α were the predominant FA in the MP14 group. After 14 days, there were significant differences between the CON14 and MP14 groups in the percentage of each FA (*p* < 0.05), with the exceptions of C4:0 and C16:1. After 14 days, the percentage of C14:0 had decreased in both groups, although the concentration of C14.0 in the MP14 was half that in the CON14 group. The percentage contributions of C16:0, C18:0, and C18:1 increased in both groups, but the relative concentration of these FA was higher in the CON14 group (by 46.2%, 38.1%, and 61.7%, respectively) than in the MP14 group. The relative concentrations of C18:2 and C18:3α had increased since the start of the experiment by 21.1% and 22.5%, respectively, in the MP14 group but had decreased by 20.9% and 44.3%, respectively, in the CON14 group. The values of C18:2 and C18:3α were higher (by 35.5% and 2.5-fold) in the MP14 group compared with the CON14 group.

The total relative concentrations of saturated, monounsaturated, and polyunsaturated FA were similar between the two groups at the beginning of the experiment. However, after 14 days, the total relative contributions of saturated and monounsaturated FA were lower by 30.2% and 15.2% in the MP14 group compared with the CON14 group, whereas the total for polyunsaturated FA were 88.6% higher in the MP14 group than in the CON14 group (Table 5). Finally, additional feeding with MP decreased biohydrogenation of PUFA in faeces. To the best of our knowledge, the impact of fermented MP feed supplement on the FA profile of calves’ faeces has not previously been studied. However, similarly to our results, Anitaş and Göncü [53] also found that palmitic (C16:0), stearic (C18:0), oleic (C18:1 n9cis), and linoleic (C18:2) acids predominated in cattle faeces. Another study reported that C14:0 and C18:2 are important for reduction in rumen methanogenesis, while C18:2 could also lead to a reduction in protozoa [54]. In our study, after 14 days of feeding, the content of C18:2 increased significantly in the MP14 group compared with the CON14 group.

### 3.7. Short-Chain Fatty Acids in the Calves’ Faeces

The quantitative SCFA profiles of the calves’ faeces before the experiment and after 14 days of feeding with fermented MP are given in Table 6. There were significant differences between the CON0 and MP0 groups in the concentrations of most of the faecal SCFA (*p* < 0.05). However, there was no significant difference between the two groups in the concentrations of acetic and butyric acids, which were the most prevalent faecal SCFA in both groups. Valeric and caproic acids were not found in the CON0 group, while the concentrations of propionic and butyric acids in the MP0 group were significantly higher (by 2-fold and 77%, respectively) than in the CON0 group. There were also significant differences between the CON14 and MP14 groups in the concentration of each SCFA (*p* < 0.05), with the exception of caproic acid. After 14 days, the predominant SCFA in the faeces of both groups were acetic, propionic, and butyric acids. The concentration of each of these acids was significantly higher in the MP14 group than in the CON14 group by 34.3%, 57.3%, and 22.3%, respectively. However, after 14 days, these predominant SCFA had increased from baseline concentrations in both groups. Acetic acid increased 4.9- and 4.6-fold in the CON14 and MP14 groups, respectively, while propionic acid increased 19.6- and 32.9-fold, respectively, and butyric acid increased 6.2- and 4.3-fold, respectively (Table 6).

In young ruminants, milk-based feeds are mainly digested in in the intestines due to their underdeveloped rumen [55]. SCFA are end products of the microbial fermentation and provide energy to the gut cells. It has been reported that branched-chain SCFA (isobutyric and isovaleric acids) are formed during utilisation of nondigested proteins and fermentation of amino acids by *Clostridium*, *Peptostreptococcus*, and *Bacteroides* in the lower gut [55]. Butyric acid improves the protective functions of the digestive tract, while propionic acid is important for T-cell production and inhibition of proinflammatory cytokines and leads to a better immune response [56]. In our study, we found a greater increase in propionic acid in the faeces of the MP14 group than in the CON14 group. The GOSs and *L. uvarum* LUHS245 in MP could contribute to the increased production of SCFA, while GOSs are also important as a nutrient source for *Bifidobacterium* and *Lactobacillus* [57]. This could explain why a higher total concentration of SCFA was found in the faeces of the MP14 group.

## 4. Conclusions

In this study, a 14-day experiment was conducted with two groups of newborn calves, the control group, and the MP group, which received an MP supplement fermented with *L. uvarum* LUHS245. After 14 days of treatment, there were no significant differences between the MP14 and CON14 groups in blood parameters, growth performance, and faecal pH. However, there were significantly higher numbers of live LAB (by 17.02%), a lower TCE (by 10.38%), and a higher content of dry matter in the faeces after 14 days in the MP group compared with the CON group (*p* < 0.05). The variety and composition of microbial species in the faeces of calves in the MP group differed significantly from the CON group in that there were more probiotic bacteria (particularly LAB) and a 1.7-fold greater variety of species. The profiles of FA (with the exceptions of C4:0 and C16:1) and VCs (with the exceptions of indole, hexanoic acid, 2-nonanone, and nonanoic acid) differed significantly between the two groups. The predominant SCFA in the faeces of both groups were acetic, propionic, and butyric acids, the concentrations of which were significantly higher in the faeces of the MP14 group compared with the CON14 group by 34.3%, 57.3%, and 22.3%, respectively. The results suggest that supplementing the feed of newborn calves with fermented MP has a positive effect on some parameters, which may potentially affect health, although blood parameters and growth performance were not influenced by the treatment.

## Figures and Tables

**Table 1:** Blood parameters and growth performance of calves fed with milk replacer only (CON group) or supplemented with fermented milk permeate (MP group).

Parameter	Day	CON	MP	*p* Day × Treat. Int.
AST (μkat/L)	Baseline	82.70 ± 54.07 ^A; a^	95.10 ± 31.23 ^A; a^	0.299
	14	43.57 ± 4.57 ^A; a^	55.57 ± 27.58 ^A; a^	
Lactates (mmol/L)	Baseline	5.27 ± 2.05 ^A; a^	4.83 ± 0.94 ^A; a^	0.411
	14	4.04 ± 2.34 ^A; a^	2.69 ± 1.91 ^A; a^	
Growth performance	Baseline	39.10 ± 3.93 ^A; a^	39.70 ± 3.77 ^A; a^	0.0001
	14	45.00 ± 5.27 ^B; a^	46.00 ± 5.09 ^B; a^	

The data are presented as mean ± standard error (*n* = 10/group). Baseline measurements were done on day 2 before the start of the feeding experiment. AST, aspartate aminotransferase; Treat. Int., treatment interaction. ^A,B^ different capitals indicate significant time-related differences (*p* < 0.05). ^a,b^ different letters indicate differences among treatments (*p* < 0.05).

**Table 2:** Faecal microbiological parameters of calves fed with milk replacer only (CON group) or supplemented with fermented milk permeate (MP group).

Microorganisms Count, log_10_ CFU/g	Day	CON	MP	*p* Day × Treat. Int.
LAB	Baseline	5.29 ± 1.89 ^A; a^	6.14 ± 1.46 ^A; a^	0.004
	14	6.14 ± 1.46 ^A; a^	7.40 ± 0.43 ^B; b^	
TCE	Baseline	6.15 ± 0.04 ^A; a^	7.23 ± 1.35 ^B; a^	0.200
	14	7.71 ± 0.55 ^A; b^	6.91 ± 0.64 ^A; a^	
TCM	Baseline	6.89 ± 1.54 ^A; a^	7.55 ± 1.25 ^A; a^	0.076
	14	8.26 ± 0.93 ^B; a^	7.71 ± 0.63 ^A; a^	
*Enterococcus faecalis*	Baseline	5.86 ± 1.12 ^A; a^	6.20 ± 0.97 ^A; a^	0.053
	14	5.04 ± 0.93 ^A; a^	5.41 ± 0.78 ^B; a^	
Y/F	Baseline	4.77 ± 1.08 ^A; a^	5.40 ± 0.92 ^A; a^	0.764
	14	5.06 ± 0.80 ^A; a^	5.35 ± 1.20 ^A; a^	

The data are presented as the mean ± standard error (*n* = 10/group). Baseline measurements were done on day 2 before the start of the feeding experiment. CFU, colony-forming units; LAB, lactic acid bacteria count; TCE, total count of enterobacteria; TCM, total count of aerobic and facultative anaerobic microorganisms; Treat. Int., treatment interaction; Y/F, yeast/fungi. ^A,B^ different capitals indicate significant time-related differences (*p* < 0.05). ^a,b^ different letters indicate differences among treatments (*p* < 0.05).

**Table 3:** The pH, dry matter, and colour coordinates of faeces in the calf group fed with milk replacer only (CON group) or supplemented with fermented milk permeate (MP group).

	Day	CON	MP	*p* Day x Treat. Int.
pH	Baseline	5.66 ± 0.18 ^A; a^	5.61 ± 0.18 ^A; a^	0.0001
	14	6.60 ± 0.28 ^B; a^	6.38 ± 0.25 ^B; a^	
Dry matter (%)	Baseline	32.28 ± 4.63 ^A; a^	33.65 ± 7.58 ^A; a^	0.0001
	14	14.90 ± 5.82 ^B; a^	22.74 ± 4.80 ^B; b^	
Texture (mJ)	Baseline	0.11 ± 0.03 ^A; a^	0.11 ± 0.03 ^A; a^	0.090
	14	0.07 ± 0.05 ^B; a^	0.11 ± 0.06 ^A; a^	
*Colour coordinates*, *NBS:*				
L*	Baseline	38.97 ± 7.30 ^A; a^	38.67 ± 9.74 ^A; a^	0.0001
	14	63.19 ± 14.56 ^B; a^	61.21 ± 11.47 ^B; a^	
a*	Baseline	1.38 ± 2.30 ^A; a^	0.19 ± 2.87 ^A; a^	0.044
	14	−1.14 ± 1.88 ^B; a^	−0.57 ± 1.01 ^A; a^	
b*	Baseline	14.31 ± 7.72 ^A; a^	11.45 ± 5.51 ^A; a^	0.959
	14	15.82 ± 10.73 ^A; a^	15.34 ± 6.32 ^A; a^	

The data are presented as the mean ± standard error (*n* = 10/group). Baseline measurements were done on day 2 before the start of the feeding experiment. L*, lightness; a*, redness or −a*, greenness; b*, yellowness or -b*, blueness; NBS, National Bureau of Standards units; Treat. Int., treatment interaction. ^A,B^ different capitals indicate significant time-related differences (*p* < 0.05). ^a,b^ different letters indicate differences among treatments (*p* < 0.05).

**Table 4:** Volatile compound percentage profiles of faeces in the calf group fed with milk replacer only (CON0 and CON14 group) or supplemented with fermented milk permeate (MP0 and MP14 group).

Volatile Compound	Calf Groups				*p*			
	CON0	CON14	MP0	MP14	CON0 × CON14	MP0 × MP14	CON0 × MP0	CON14 × MP14
Acetic acid	3.71 ± 0.31	2.24 ± 0.18	1.950 ± 0.11	0.640 ± 0.050	0.003	0.001	0.004	0.002
Acetoin	7.32 ± 0.62	nd	1.40 ± 0.25	nd	0.002	0.011	-	0.001
Propanoic acid	nd	2.62 ± 0.18	nd	2.20 ± 0.14	0.002	0.001	-	0.003
Butanoic acid	9.53 ± 0.59	32.39 ± 2.84	22.13 ± 1.93	24.94 ± 2.10	0.003	0.001	0.004	0.003
Butanoic acid, 3-methyl-	2.36 ± 0.24	2.33 ± 0.18	0.59 ± 0.04	2.21 ± 0.22	0.564	0.004	0.004	0.032
Butyric acid <2-methyl->	4.24 ± 0.38	4.27 ± 0.29	1.09 ± 0.09	2.65 ± 0.17	0.603	0.001	0.003	0.002
2-Heptanone	6.77 ± 0.34	nd	2.54 ± 0.24	nd	0.001	0.003	-	0.0001
Pentanoic acid	0.22 ± 0.02	5.87 ± 0.41	0.171 ± 0.010	2.40 ± 0.15	0.002	0.001	0.009	0.002
Hexanoic acid	2.51 ± 0.18	1.46 ± 0.11	3.89 ± 0.28	1.62 ± 0.11	0.001	0.002	0.002	0.321
Phosphonic acid, (*p*-hydroxyphenyl)-	nd	0.478 ± 0.040	nd	3.78 ± 0.03	0.002	0.0001	-	0.0001
1-Hexanol, 2-ethyl-	5.99 ± 0.35	1.72 ± 0.21	1.49 ± 0.15	3.06 ± 0.28	0.0001	0.002	0.001	0.001
2-Propyl-1-pentanol	0.011 ± 0.001	nd	9.61 ± 0.46	nd	0.003	0.001	0.001	-
*p*-Cresol	0.992 ± 0.070	21.98 ± 0.93	1.53 ± 0.17	26.56 ± 1.48	0.001	0.001	0.012	0.005
2-Nonanone	9.64 ± 0.74	0.207 ± 0.021	6.47 ± 0.43	0.173 ± 0.021	0.002	0.001	0.003	0.145
Phenethyl alcohol	1.95 ± 0.16	0.436 ± 0.05	2.35 ± 0.19	0.627 ± 0.050	0.002	0.002	0.002	0.0001
Octanoic acid	7.43 ± 0.51	0.521 ± 0.040	5.99 ± 0.47	0.727 ± 0.060	0.002	0.002	0.0001	0.003
Nonanoic acid	12.17 ± 1.23	1.13 ± 0.56	10.39 ± 1.23	0.921 ± 0.110	0.001	0.005	0.0001	0.515
Indole	6.87 ± 0.65	11.90 ± 0.92	12.24 ± 0.87	12.16 ± 1.18	0.001	0.685	0.001	0.231
*n*-Decanoic acid	3.10 ± 0.32	0.663 ± 0.05	2.98 ± 0.28	0.639 ± 0.040	0.037	0.003	0.127	0.053

The data are presented as the mean ± standard error (*n* = 10/group). 0, Baseline measurements; 14, after 14 days of feeding. CON, calves fed with milk replacer only; MP, calves fed with milk replacer and supplemented with fermented milk permeate; nd, not detected.

**Table 5:** Fatty acid percentage profiles of faeces in the calf group fed with milk replacer only (CON0 and CON14 group) or supplemented with fermented milk permeate (MP0 and MP14 group).

Fatty Acid		Calf Groups				*p*			
		CON0	CON14	MP0	MP14	CON0 × CON14	MP0 × MP14	CON0 × MP0	CON14 × MP14
C4:0	Butyric	nd	0.051 ± 0.004	Nd	0.064 ± 0.005	0.002	0.002	-	0.129
C6:0	Caproic	nd	0.072 ± 0.006	Nd	nd	0.002	-	-	0.002
C8:0	Caprylic	0.213 ± 0.038	0.047 ± 0.003	0.870 ± 0.080	nd	0.014	0.003	0.001	0.001
C10:0	Capric	2.84 ± 0.19	0.268 ± 0.003	4.06 ± 0.31	0.009 ± 0.001	0.002	0.002	0.003	0.0001
C11:0	Undecylic	0.096 ± 0.021	nd	0.400 ± 0.012	nd	0.016	0.0001	0.0001	-
C12:0	Lauric	2.15 ± 0.22	0.491 ± 0.004	3.00 ± 0.18	0.198 ± 0.08	0.006	0.0001	0.001	0.022
C13:0	Tridecylic	0.114 ± 0.024	nd	0.387 ± 0.024	nd	0.014	0.001	-	-
C14:0	Myristic	14.7 ± 1.1	4.17 ± 0.15	15.1 ± 1.2	2.06 ± 0.18	0.003	0.002	0.02	0.0001
C14:1	Myristoleic	0.045 ± 0.011	0.075 ± 0.006	0.042 ± 0.004	0.003 ± 0.001	0.009	0.002	0.535	0.002
C15:0	Pentadecylic	0.429 ± 0.023	0.150 ± 0.002	0.715 ± 0.021	0.234 ± 0.011	0.002	0.0001	0.0001	0.004
C16:0	Palmitic	16.8 ± 1.5	26.9 ± 2.5	13.09 ± 0.92	18.4 ± 0.1	0.049	0.008	0.008	0.03
C16:1	Palmitoleic	0.23 ± 0.02	0.529 ± 0.021	0.384 ± 0.023	0.529 ± 0.035	0.0001	0.002	0.0001	1
C17:0	Margaric	0.091 ± 0.008	0.158 ± 0.010	0.172 ± 0.009	0.426 ± 0.027	0.0001	0.002	0.0001	0.001
C17:1	Heptadecenoic	0.016 ± 0.003	0.053 ± 0.004	0.021 ± 0.003	0.019 ± 0.002	0.0001	0.074	0.512	0.001
C18:0	Stearic	8.12 ± 0.09	14.5 ± 0.3	6.17 ± 0.35	10.5 ± 0.3	0.0001	0.0001	0.006	0.0001
C18:1 (cis,trans)	Oleic, elaidic	18.2 ± 1.2	29.6 ± 2.6	16.6 ± 0.95	18.3 ± 0.2	0.005	0.059	0.008	0.015
C18:2 cis	Linoleic acid	13.9 ± 1.6	11.0 ± 0.1	12.3 ± 0.62	14.9 ± 0.1	0.079	0.013	0.106	0.004
C18:3 γ	γ-Linolenic	0.167 ± 0.012	nd	0.695 ± 0.032	0.316 ± 0.013	0.002	0.001	0.0001	0.001
C18:3 α	α-Linolenic	16.5 ± 1.4	9.19 ± 0.81	18.7 ± 0.91	22.9 ± 0.2	0.002	0.009	0.016	0.001
C20:0	Arachidic	0.328 ± 0.03	0.043 ± 0.003	0.214 ± 0.017	0.585 ± 0.028	0.003	0.0001	0.004	0.001
C20:1 ω9	Gondoic	2.6 ± 0.2	0.429 ± 0.012	2.17 ± 0.53	6.79 ± 0.24	0.002	0.001	0.153	0.0001
C20:2 ω6	Eicosadienoic	0.226 ± 0.015	1.11 ± 0.09	0.146 ± 0.031	0.913 ± 0.031	0.002	0.0001	0.013	0.029
C20:3 ω6	Dihomo-γ-linolenic (DGLA)	0.047 ± 0.009	nd	0.053 ± 0.004	0.124 ± 0.018	0.012	0.013	0.173	0.007
C20:4 ω6	Arachidonic	0.115 ± 0.010	0.053 ± 0.004	0.071 ± 0.006	0.236 ± 0.014	0.003	0.001	0.003	0.001
C20:3 ω3	Eicosatrienoic	0.203 ± 0.018	nd	0.150 ± 0.014	0.912 ± 0.024	0.003	0.0001	0.002	0.0001
C20:5 ω3	Eicosapentaenoic (EPA)	0.025 ± 0.003	0.011 ± 0.001	nd	nd	0.007	-	0.005	0.003
C22:0	Behenic	0.085 ± 0.009	nd	nd	0.216 ± 0.017	0.004	0.002	0.004	0.002
C22:1 ω9	Erucic	1.44 ± 0.14	1.10 ± 0.08	4.37 ± 0.38	1.30 ± 0.15	0.01	0.002	0.002	0.038
C24:0	Lignoceric	0.228 ± 0.015	nd	0.146 ± 0.021	nd	0.001	0.007	0.002	-
C22:6 ω3	Docosahexaenoic (DHA)	0.037 ± 0.002	nd	nd	nd	0.001	-	0.001	-
Total saturated		46.194	46.85	44.324	32.692	-	-	-	-
Total monounsaturated		22.531	31.786	23.587	26.941	-	-	-	-
Total polyunsaturated		31.22	21.364	32.115	40.301	-	-	-	-

The data are presented as mean ± SE (*n* = 10/group). 0, baseline measurements; 14, after 14 days of feeding. nd, not detected.

**Table 6:** Short-chain fatty acids (SCFA) in faeces in the calf group fed with milk replacer only (CON0 and CON14 group) or supplemented with fermented milk permeate (MP0 and MP14 group).

SCFA (mmol/kg)	Calf Groups				*p*			
	CON0	CON14	MP0	MP14	CON0 × CON14	MP0 × MP14	CON0 × MP0	CON14 × MP14
Acetic	13.32 ± 4.89	65.01 ± 13.59	19.11 ± 3.45	87.31 ± 11.29	0.009	0.015	0.352	0.004
Propionic	0.461 ± 0.053	20.07 ± 4.23	0.959 ± 0.142	31.58 ± 4.26	0.015	0.006	0.010	0.0001
Isobutyric	0.144 ± 0.053	1.13 ± 0.20	0.013 ± 0.004	2.82 ± 0.48	0.007	0.009	0.044	0.009
Butyric	2.60 ± 0.51	16.20 ± 2.21	4.60 ± 0.57	19.82 ± 3.49	0.005	0.855	0.384	0.039
Isovaleric	0.242 ± 0.031	1.37 ± 0.42	0.074 ± 0.014	4.02 ± 0.31	0.037	0.002	0.003	0.001
Valeric	nd	0.874 ± 0.076	0.024 ± 0.005	0.618 ± 0.082	-	0.006	-	0.0001
Caproic	nd	0.128 ± 0.34	0.121 ± 0.036	0.078 ± 0.011	-	0.023	-	0.064
*Total*	16.767	104.782	24.901	146.246	-	-	-	-

The data are presented as the mean ± standard error (*n* = 10/group). 0, baseline measurements; 14, after 14 days of feeding. nd, not detected.

## Data Availability

All the data is given in Supplementary Files.

## Supplementary Materials

The following are available online at https://www.mdpi.com/2076-2615/11/9/2544/s1, Table S1: pH, total titratable acidity (TTA), and total lactic acid bacteria (LAB) count in the milk permeate (MP) samples; Table S2: Antimicrobial activities of the nonfermented and fermented milk permeate (MP) against 15 pathogenic and opportunistic microbial bacteria in liquid medium (+ indicates pathogen growth;—indicates that pathogen growth was not established); Table S3: Galactooligosaccharides (GOS) (mg_GOS_/100 mL_sample_) concentration in nonfermented and fermented milk permeate (MP) samples; Table S4: Bacterial genera in the faeces of calves fed with fermented milk permeate (MP group) after 14 days of the experiment; Table S5: Bacterial genera in the faeces of calves fed with milk replacer only (CON group) after 14 days of the experiment; Table S6: Bacterial species in the faeces of calves fed with fermented milk permeate (MP group) after 14 days of the experiment; Table S7: Bacterial species in the faeces of calves fed with milk replacer only (CON group) after 14 days of the experiment; Table S8: Volatile compound percentage profiles of faeces in the control (CON) and supplemented with fermented milk permeate (MP) groups.
