# Supplementary material for: Supplement Based on Fermented Milk Permeate for Feeding Newborn Calves: Influence on Blood, Growth Performance, and Faecal Parameters, including Microbiota, Volatile Compounds, and Fatty and Organic Acid Profiles
Source: Animals (Basel). 2021 Aug 30;11(9):2544. doi: 10.3390/ani11092544 (PMC8466287; doi:10.3390/ani11092544)
Supplement: Supplementary file 1 [file animals-11-02544-s001.zip › animals-1307689-supplementary.pdf]

Table S1. pH, total titratable acidity (TTA), and total lactic acid bacteria (LAB) count in the milk permeate (MP) samples.

Milk Permeate	pH	TTA (°N)	LAB viable counts (log_{10} CFU/mL)
MPNF	5.88 ±0.8	3.0 ±0.14 [a]	nd
$MP_{LUHS245}$	3.97 ±0.07	9.5 ±0.29	8.68 ±0.39

LAB—lactic acid bacteria; CFU—colony-forming units; TTA—total titratable acidity; nd—not detected. MP—milk permeate; $MP_{LUHS245}$—fermented with LUHS245 (*L. uvarum);* MPNF—non-fermented.

Table S2. Antimicrobial activities of the nonfermented and fermented milk permeate (MP) against 15 pathogenic and opportunistic microbial bacterial in liquid medium (+ indicates pathogen growth; - indicates that pathogen growth was not established).

	Growth (+) or Growth Absence (-) of Pathogenic and Opportunistic Bacteria															
	Pathogenic and Opportunistic Bacterial Strains															
**Samples**	***1***	***2***	***3***	***4***	***5***	***6***	***7***	***8***	***9***	***10***	***11***	***12***	***13***	***14***	***15***	Number of the inhibited pathogens
$MP_{LUHS245}$	-	-	-	-	-	-	+	+	-	-	-	-	-	-	-	13
MPNF	+	+	+	+	-	-	+	+	+	+	+	+	+	+	+	0
Pathogen control	+	+	+	+	+	+	+	+	+	+	+	+	+	+	+	-
LAB control	+	+	+	+	+	+	+	+	+	+	+	+	+	+	+	-

MP—milk permeate; $MP_{LUHS245}$—fermented with LUHS245 (*L. uvarum);* 1 - *Klebsiella pneumonia; 2 - Salmonella enterica; 3 - Pseudomonas aeruginosa; 4 - Acinetobacter baumannii; 5 - Proteus mirabilis; 6* - MRSA M87fox; 7 - *Enterococcus faecalis; 8 - Enterococcus faecium; 9 - Bacillus cereus; 10 - Streptococcus mutans; 11 - Enterobacter cloacae; 12 - Citrobacter freundii; 13 - Streptococcus epidermis; 14 - Staphylococcus haemolyticus; 15 - Pasteurella multocida.*

Table S3. Galactooligosaccharides (GOS) ($mg_{GOS}/100mL_{sample}$) concentration in nonfermented and fermented milk permeate (MP) samples.

Sample	G2, $mg_{GOS}/100mL$	G3, $mg_{GOS}/100mL$
$MP_{LUHS245}$	8.2	1.8
MP_{non}	nd	nd

MP_{non} – nonfermented milk permeate; $MP_{LUHS245}$—fermented with LUHS245 (*L. uvarum);* G2—galactobiose; G3—galactotriose; nd—not detected.

Table S4. Bacterial genera in the feces of calves fed with fermented milk permeate (MP group) after 14 days of the experiment.

Bacterial genera in MP group after experiment	Number of reads	Relative abundance
Lactobacillus	10185	36.03%
Bifidobacterium	2590	9.16%
Bacteroides	2342	8.28%
Blautia	2209	7.81%
Tyzzerella	2176	7.69%
Erysipelatoclostridium	1490	5.27%
Escherichia	1353	4.78%
Holdemanella	505	1.78%
Psychrobacter	502	1.77%
Lachnoclostridium	453	1.6%
Dorea	448	1.58%
Fusobacterium	338	1.19%
Unclassified	323	1.14%
Gemmiger	265	0.93%
Phascolarctobacterium	207	0.73%
Coprobacter	204	0.72%
Butyricicoccus	203	0.71%
Ruminococcus	202	0.71%
unclassified Fusobacteria	157	0.55%
Actinomyces	143	0.5%
Arthrobacter	109	0.38%
Faecalibacterium	107	0.37%
Gallibacterium	98	0.34%
Akkermansia	96	0.33%
Eubacterium	88	0.31%
Sutterella	85	0.3%
Carnobacterium	77	0.27%
Mannheimia	64	0.22%
Kluyvera	63	0.22%
Pseudoalteromonas	61	0.21%
Mobilitalea	59	0.2%

Streptococcus	52	0.18%
Anaerobacterium	50	0.17%
Enterococcus	50	0.17%
Coprococcus	49	0.17%
Roseburia	42	0.14%
Clostridium	41	0.14%
Butyrivibrio	31	0.1%
Bacillus	26	0.09%
Glutamicibacter	26	0.09%
Parabacteroides	25	0.08%
Alistipes	24	0.08%
Staphylococcus	22	0.07%
Eggerthella	22	0.07%
Flavonifractor	22	0.07%
Shigella	20	0.07%
Faecalicoccus	20	0.07%
unclassified Bacteroidales	19	0.06%
Granulicatella	17	0.06%
Subdoligranulum	17	0.06%
Peptostreptococcus	17	0.06%
Vibrio	15	0.05%
Anaerotaenia	15	0.05%
Ruminiclostridium	15	0.05%
Hespellia	15	0.05%
Butyricimonas	14	0.04%
Acetivibrio	14	0.04%
Anaerocolumna	14	0.04%
Herbinix	13	0.04%
Pseudomonas	13	0.04%
Hungatella	13	0.04%
Corynebacterium	12	0.04%
Fusicatenibacter	12	0.04%
Aerococcus	12	0.04%
Odoribacter	12	0.04%

Sphingobacterium	11	0.03%
Oceanisphaera	9	0.03%
Howardella	9	0.03%
Aerosphaera	8	0.02%
Anaerostipes	8	0.02%
Kocuria	8	0.02%
Solobacterium	8	0.02%
unclassified Bacteroidaceae	8	0.02%
Photorhabdus	8	0.02%
Pseudarthrobacter	7	0.02%
Leuconostoc	7	0.02%
Vagococcus	7	0.02%
Salmonella	7	0.02%
Casaltella	6	0.02%
Prevotella	6	0.02%
unclassified Erysipelotrichaceae	6	0.02%
Fournierella	6	0.02%
unclassified Gammaproteobacteria	6	0.02%
Kosakonia	6	0.02%
Robinsoniella	4	0.01%
Muribacter	4	0.01%
Falcatimonas	4	0.01%
Ruthenibacterium	4	0.01%
Pseudobutyrivibrio	4	0.01%
Flintibacter	4	0.01%
Parasutterella	4	0.01%
Oribacterium	4	0.01%
Pediococcus	4	0.01%
Murimonas	4	0.01%
unclassified Lactobacillaceae	3	0.01%
Erysipelothrix	3	0.01%
Salinicoccus	3	0.01%
Trueperella	3	0.01%

Serratia	3	0.01%
Paraprevotella	3	0.01%
Jeotgalicoccus	3	0.01%
Amphritea	3	0.01%
Petrimonas	2	0%
Myroides	2	0%
Streptomyces	2	0%
unclassified Lachnospiraceae	2	0%
unclassified Clostridiales	2	0%
Dietzia	2	0%
Natranaerovirga	2	0%
Eisenbergiella	2	0%
Veillonella	2	0%
Atopobium	2	0%
Citrobacter	2	0%
Alloprevotella	1	0%
Oscillibacter	1	0%
Proteinivorax	1	0%
Metabacterium	1	0%
Intestinimonas	1	0%
Christensenella	1	0%
Cutibacterium	1	0%
Microbacterium	1	0%
Methylobacterium	1	0%
Paludibacter	1	0%
Renibacterium	1	0%
Lactonifactor	1	0%
Luteococcus	1	0%
unclassified Alphaproteobacteria	1	0%
Pseudoclavibacter	1	0%
Stomatobaculum	1	0%
Lactococcus	1	0%
Bariatricus	1	0%

unclassified Deltaproteobacteria	1	0%
Neptuniibacter	1	0%
Nocardioides	1	0%
Jeotgalibaca	1	0%
Proteiniborus	1	0%
Moraxella	1	0%
Shewanella	1	0%
Brevundimonas	1	0%
Sphingomonas	1	0%
Catabacter	1	0%
Abyssivirga	1	0%
Peptoclostridium	1	0%
Vallitalea	1	0%
Enterobacter	1	0%
Leucobacter	1	0%
Agathobacter	1	0%
Anaerofustis	1	0%
Frondihabitans	1	0%
Salirhabdus	1	0%
Bulleidia	1	0%
unclassified Pasteurellaceae	1	0%
Ethanoligenens	1	0%
Brassicibacter	1	0%
Lachnoanaerobaculum	1	0%
Neorhizobium	1	0%
Haemophilus	1	0%
Acidaminococcus	1	0%
Zhihengliuella	1	0%
Izhakiella	1	0%
Oscillospira	1	0%
Rhizobium	1	0%
Limnobacter	1	0%
Turicibacter	1	0%
Paeniglutamicibacter	1	0%

Shuttleworthia	1	0%
Rhodococcus	1	0%
Rhodobacter	1	0%

Table S5. Bacterial genera in the feces of calves fed with milk replacer only (CON group) after 14 days of the experiment.

Bacterial genera in CON group after experiment	Number of reads	Relative abundance
Blautia	10978	40.7%
Tyzzerella	3446	12.77%
Bacteroides	3249	12.04%
Butyricicoccus	2864	10.61%
Ruminococcus	1012	3.75%
Erysipelatoclostridium	974	3.61%
Faecalibacterium	835	3.09%
Bifidobacterium	731	2.71%
Lactobacillus	631	2.33%
Escherichia	310	1.14%
Prevotella	276	1.02%
Lachnoclostridium	248	0.91%
Unclassified	181	0.67%
Dorea	120	0.44%
Coprococcus	108	0.4%
Howardella	97	0.35%
Streptococcus	95	0.35%
Clostridium	84	0.31%
Hespellia	65	0.24%
Holdemanella	58	0.21%
Actinomyces	53	0.19%
Eubacterium	47	0.17%
Murimonas	40	0.14%
Anaerostipes	38	0.14%
Alloprevotella	30	0.11%
Kluyvera	29	0.1%
unclassified Bacteroidaceae	25	0.09%

Corynebacterium	23	0.08%
Pseudobutyrivibrio	21	0.07%
Butyrivibrio	19	0.07%
Coprobacillus	17	0.06%
Mobilitalea	16	0.05%
Roseburia	15	0.05%
Anaerococcus	14	0.05%
Casaltella	12	0.04%
Shigella	11	0.04%
unclassified Lachnospiraceae	11	0.04%
Faecalicoccus	10	0.03%
Pseudoflavonifractor	8	0.02%
unclassified Bacteroidales	8	0.02%
Sutterella	8	0.02%
Lactonifactor	7	0.02%
Ruminiclostridium	7	0.02%
Peptostreptococcus	6	0.02%
Ewingella	6	0.02%
Parabacteroides	5	0.01%
Lactococcus	5	0.01%
Gemmiger	5	0.01%
Kosakonia	5	0.01%
Eisenbergiella	5	0.01%
Flavonifractor	5	0.01%
Anaerocolumna	4	0.01%
Stomatobaculum	4	0.01%
Sharpea	4	0.01%
Hungatella	4	0.01%
Catenibacterium	3	0.01%
Flintibacter	3	0.01%
Fournierella	3	0.01%
Robinsoniella	2	0%
Parvimonas	2	0%
Staphylococcus	2	0%

Comamonas	2	0%
unclassified Erysipelotrichaceae	2	0%
Phascolarctobacterium	2	0%
unclassified Clostridiales	2	0%
Serratia	2	0%
Photorhabdus	2	0%
unclassified Clostridiales Family XIII. Incertae Sedis	2	0%
Oribacterium	2	0%
Anaerobium	2	0%
Wautersiella	2	0%
Johnsonella	1	0%
Lachnoanaerobaculum	1	0%
Gallibacterium	1	0%
Enterobacter	1	0%
Vallitalea	1	0%
Citrobacter	1	0%
Anaerotaenia	1	0%
unclassified Gammaproteobacteria	1	0%
Pantoea	1	0%
Aquicella	1	0%
Achromobacter	1	0%
Gottschalkia	1	0%
Anaerobacterium	1	0%
Mogibacterium	1	0%
unclassified Lactobacillaceae	1	0%
Afipia	1	0%
Drancourtella	1	0%
Peptoniphilus	1	0%
Fusicatenibacter	1	0%
Salmonella	1	0%
Peptococcus	1	0%
Enterococcus	1	0%
Lachnospira	1	0%

Turicibacter	1	0%
Lautropia	1	0%
Ralstonia	1	0%
Flavobacterium	1	0%
unclassified Prevotellaceae	1	0%
Subdoligranulum	1	0%
Falcatimonas	1	0%
unclassified Peptostreptococcaceae	1	0%
Acetobacter	1	0%
Bariatricus	1	0%
Crassaminicella	1	0%
Natranaerovirga	1	0%
Sulfitobacter	1	0%
unclassified Clostridia	1	0%
Intestinimonas	1	0%
Bilophila	1	0%
Dysgonomonas	1	0%
Acetivibrio	1	0%
Cutibacterium	1	0%
Bacillus	1	0%
Klebsiella	1	0%

Table S6. Bacterial species in the feces of calves fed with fermented milk permeate (MP group) after 14 days of the experiment.

Bacterial species in MP group after experiment	Number of reads	Relative abundance
Lactobacillus amylovorus	6032	21.34%
Bifidobacterium longum	2115	7.48%
Tyzzerella nexilis	2014	7.12%
Lactobacillus johnsonii	1882	6.65%
Erysipelatoclostridium ramosum	1486	5.25%
Ruminococcus torques	1468	5.19%
Bacteroides fragilis	1327	4.69%

Escherichia coli	1319	4.66%
Lactobacillus reuteri	1077	3.81%
Ruminococcus gnavus	697	2.46%
Holdemanella biformis	505	1.78%
Dorea formicigenerans	433	1.53%
Unclassified	323	1.14%
Gemmiger formicilis	265	0.93%
Fusobacterium russii	240	0.84%
Bacteroides coprophilus	222	0.78%
Phascolarctobacterium succinatutens	207	0.73%
Coprobacter fastidiosus	204	0.72%
Butyricicoccus pullicaecorum	203	0.71%
Lactobacillus jensenii	184	0.65%
Bifidobacterium pseudocatenulatum	181	0.64%
Ruminococcus faecis	177	0.62%
Bacteroides denticanum	172	0.6%
Psychrobacter phenylpyruvicus	169	0.59%
Bacteroides thetaiotaomicron	163	0.57%
Clostridium lactatifermentans	159	0.56%
Clostridium rectum	157	0.55%
Lactobacillus delbrueckii	152	0.53%
Bifidobacterium breve	143	0.5%
Lactobacillus gasseri	138	0.48%
Clostridium aldenense	128	0.45%
Lactobacillus crispatus	118	0.41%
Lactobacillus hominis	108	0.38%
Faecalibacterium prausnitzii	107	0.37%
Akkermansia muciniphila	96	0.33%
Gallibacterium anatis	95	0.33%
Eubacterium fissicatena	91	0.32%
Sutterella stercoricanis	83	0.29%
Lactobacillus helveticus	82	0.29%
Arthrobacter psychrolactophilus	80	0.28%
Psychrobacter psychrophilus	78	0.27%

Actinomyces hyovaginalis	74	0.26%
Fusobacterium mortiferum	72	0.25%
Clostridium symbiosum	72	0.25%
Psychrobacter cibarius	72	0.25%
Bacteroides pyogenes	68	0.24%
Bacteroides uniformis	63	0.22%
Lactobacillus acidophilus	62	0.21%
Kluyvera georgiana	59	0.2%
Mobilitalea sibirica	59	0.2%
Mannheimia caviae	58	0.2%
Bacteroides coprocola	56	0.19%
Bacteroides vulgatus	55	0.19%
Bacteroides heparinolyticus	54	0.19%
Psychrobacter maritimus	51	0.18%
Carnobacterium maltaromaticum	51	0.18%
Actinomyces cardiffensis	51	0.18%
Anaerobacterium chartisolvens	50	0.17%
Lactobacillus mucosae	49	0.17%
Psychrobacter faecalis	47	0.16%
Lactobacillus kitasatonis	43	0.15%
Coprococcus comes	43	0.15%
Bifidobacterium catenulatum	42	0.14%
Eubacterium limosum	42	0.14%
Lactobacillus sakei	41	0.14%
Clostridium clostridioforme	40	0.14%
Clostridium saccharolyticum	38	0.13%
Bifidobacterium kashiwanohense	32	0.11%
Lactobacillus taiwanensis	31	0.1%
Butyrivibrio fibrisolvens	31	0.1%
Escherichia albertii	30	0.1%
Bacteroides salanitronis	29	0.1%
Eubacterium contortum	29	0.1%
Pseudoalteromonas atlantica	28	0.09%
Bacteroides helcogenes	27	0.09%

unclassified Bifidobacterium	26	0.09%
Lactobacillus pontis	25	0.08%
Glutamicibacter nicotianae	24	0.08%
Streptococcus lutetiensis	23	0.08%
Bacteroides finegoldii	23	0.08%
Eggerthella lenta	22	0.07%
Bacillus pumilus	22	0.07%
Flavonifractor plautii	22	0.07%
Blautia producta	22	0.07%
Eubacterium oxidoreducens	21	0.07%
Psychrobacter celer	21	0.07%
unclassified Lactobacillus	21	0.07%
Faecalicoccus pleomorphus	20	0.07%
Psychrobacter arcticus	19	0.06%
Eubacterium ruminantium	19	0.06%
Shigella dysenteriae	18	0.06%
Parabacteroides distasonis	17	0.06%
Ruminococcus bromii	17	0.06%
unclassified Granulicatella	17	0.06%
Subdoligranulum variabile	17	0.06%
Alistipes shahii	16	0.05%
Roseburia faecis	16	0.05%
Bacteroidales oral	16	0.05%
Bacteroides nordii	16	0.05%
Blautia stercoris	16	0.05%
Clostridium fusiformis	15	0.05%
Hespellia porcina	15	0.05%
Bacteroides plebeius	15	0.05%
Enterococcus avium	15	0.05%
Bifidobacterium scardovii	15	0.05%
Streptococcus gallolyticus	15	0.05%
Anaerotaenia torta	15	0.05%
Lactobacillus curvatus	14	0.04%
Roseburia hominis	14	0.04%

Lactobacillus hamsteri	14	0.04%
Herbinix luporum	13	0.04%
Bacteroides salyersiae	13	0.04%
Candidatus Dorea	13	0.04%
Anaerocolumna xylanovorans	13	0.04%
Roseburia inulinivorans	12	0.04%
Vibrio rumoiensis	12	0.04%
unclassified Fusobacterium	12	0.04%
Enterococcus faecalis	12	0.04%
Odoribacter splanchnicus	12	0.04%
Fusicatenibacter saccharivorans	12	0.04%
Clostridium populeti	12	0.04%
Hungatella hathewayi	11	0.03%
Psychrobacter sanguinis	11	0.03%
Lactobacillus antri	11	0.03%
Lactobacillus fuchuensis	11	0.03%
Clostridium glycyrrhizinilyticum	11	0.03%
Psychrobacter submarinus	11	0.03%
Bacteroides acidifaciens	11	0.03%
Lactobacillus rodentium	10	0.03%
Pseudoalteromonas paragorgicola	10	0.03%
Clostridium asparagiforme	9	0.03%
Lactobacillus gallinarum	9	0.03%
Howardella ureilytica	9	0.03%
Aerococcus viridans	9	0.03%
Lactobacillus fermentum	9	0.03%
Sphingobacterium faecium	9	0.03%
Enterococcus cecorum	8	0.02%
Lactobacillus graminis	8	0.02%
Psychrobacter marincola	8	0.02%
unclassified Bacteroidaceae	8	0.02%
Aerosphaera taetra	8	0.02%
Anaerostipes butyraticus	8	0.02%
Lactobacillus vaginalis	8	0.02%

Bacteroides caccae	8	0.02%
Clostridium oroticum	8	0.02%
Solobacterium moorei	8	0.02%
Acetivibrio ethanolgignens	8	0.02%
Photorhabdus luminescens	8	0.02%
Lactobacillus oryzae	7	0.02%
Clostridium butyricum	7	0.02%
Butyricimonas paravirosa	7	0.02%
Carnobacterium alterfunditum	7	0.02%
Staphylococcus equorum	7	0.02%
Clostridium perfringens	7	0.02%
Lactobacillus frumenti	7	0.02%
Peptostreptococcus russellii	7	0.02%
Salmonella enterica	7	0.02%
Carnobacterium viridans	6	0.02%
Mannheimia varigena	6	0.02%
Kocuria kristinae	6	0.02%
Clostridium scindens	6	0.02%
Arthrobacter alpinus	6	0.02%
Coprococcus eutactus	6	0.02%
Kosakonia sacchari	6	0.02%
Peptostreptococcus stomatis	6	0.02%
Lactobacillus kefiranofaciens	6	0.02%
Casaltella massiliensis	6	0.02%
Arthrobacter humicola	6	0.02%
Clostridium hylemonae	6	0.02%
unclassified Acetivibrio	6	0.02%
Pseudomonas fragi	6	0.02%
Arthrobacter stackebrandtii	6	0.02%
Fournierella massiliensis	6	0.02%
Actinomyces haliotis	6	0.02%
Enterococcus faecium	6	0.02%
Leuconostoc carnosum	6	0.02%
Clostridium viride	6	0.02%

Staphylococcus vitulinus	5	0.01%
Streptococcus equinus	5	0.01%
Bifidobacterium pullorum	5	0.01%
Bifidobacterium saguini	5	0.01%
Lactobacillus amylolyticus	5	0.01%
unclassified Erysipelotrichaceae	5	0.01%
Arthrobacter pascens	5	0.01%
Alistipes putredinis	5	0.01%
Psychrobacter namhaensis	5	0.01%
Staphylococcus sciuri	5	0.01%
Lactobacillus psittaci	5	0.01%
Bacteroides xylanisolvens	5	0.01%
Pseudomonas fluorescens	5	0.01%
Oceanisphaera ostreae	5	0.01%
Ruminococcus gauvreauii	5	0.01%
Actinomyces nasicola	5	0.01%
Falcatimonas natans	4	0.01%
Robinsoniella peoriensis	4	0.01%
Parasutterella excrementihominis	4	0.01%
gamma proteobacterium	4	0.01%
Eubacterium coprostanoligenes	4	0.01%
Bifidobacterium stellenboschense	4	0.01%
Murimonas intestini	4	0.01%
Prevotella genomosp.	4	0.01%
Bifidobacterium biavatii	4	0.01%
Pseudoalteromonas spongiae	4	0.01%
Parabacteroides merdae	4	0.01%
Vagococcus lutrae	4	0.01%
Kluyvera ascorbata	4	0.01%
Lactobacillus oligofermentans	4	0.01%
Bacteroides faecichinchillae	4	0.01%
Carnobacterium divergens	4	0.01%
Fusobacterium varium	4	0.01%
Fusobacterium ulcerans	4	0.01%

Arthrobacter psychrochitiniphilus	4	0.01%
Ruthenibacterium lactatiformans	4	0.01%
Muribacter muris	4	0.01%
Oribacterium sinus	4	0.01%
Clostridium cellobioparum	4	0.01%
Flintibacter butyricus	4	0.01%
Streptococcus danieliae	4	0.01%
Butyricimonas faecihominis	4	0.01%
Peptostreptococcus anaerobius	4	0.01%
Corynebacterium casei	4	0.01%
unclassified Bacteroidales	3	0.01%
Fusobacterium necrogenes	3	0.01%
Pseudoalteromonas piscicida	3	0.01%
Jeotgalicoccus halotolerans	3	0.01%
Psychrobacter fozii	3	0.01%
Carnobacterium gallinarum	3	0.01%
unclassified Bacteroides	3	0.01%
Salinicoccus halodurans	3	0.01%
Clostridium neopropionicum	3	0.01%
Corynebacterium provencense	3	0.01%
Amphritea ceti	3	0.01%
Pseudobutyrivibrio xylanivorans	3	0.01%
unclassified Lactobacillaceae	3	0.01%
Bifidobacterium adolescentis	3	0.01%
Lactobacillus panis	3	0.01%
Gallibacterium salpingitidis	3	0.01%
Actinomyces suimastitidis	3	0.01%
Paraprevotella clara	3	0.01%
Clostridium saccharogumia	3	0.01%
Butyricimonas virosa	3	0.01%
Parabacteroides goldsteinii	3	0.01%
Oceanisphaera profunda	3	0.01%
Bifidobacterium bombi	2	0%
Escherichia fergusonii	2	0%

unclassified Erysipelothrix	2	0%
Escherichia marmotae	2	0%
Bacteroides faecis	2	0%
Pseudoalteromonas aliena	2	0%
Eisenbergiella tayi	2	0%
Psychrobacter glacincola	2	0%
Bifidobacterium lemurum	2	0%
Clostridium thermosuccinogenes	2	0%
Enterococcus devriesei	2	0%
Pediococcus argentinicus	2	0%
Bifidobacterium gallicum	2	0%
Bifidobacterium choerinum	2	0%
Serratia marcescens	2	0%
unclassified Gammaproteobacteria	2	0%
Lactobacillus tucceti	2	0%
Kocuria rhizophila	2	0%
Pseudarthrobacter defluvii	2	0%
Myroides guanonis	2	0%
Blautia schinkii	2	0%
Trueperella bernardiae	2	0%
Corynebacterium callunae	2	0%
Pseudoalteromonas arctica	2	0%
Pseudarthrobacter oxydans	2	0%
Carnobacterium funditum	2	0%
Fusobacterium nucleatum	2	0%
Alistipes senegalensis	2	0%
Pseudoalteromonas ruthenica	2	0%
Enterococcus lemanii	2	0%
Sutterella parvirubra	2	0%
Psychrobacter jeotgali	2	0%
Citrobacter freundii	2	0%
Eubacterium ramulus	2	0%
Carnobacterium jeotgali	2	0%
Staphylococcus lentus	2	0%

Bifidobacterium merycicum	2	0%
Hungatella effluvii	2	0%
unclassified Ruminococcus	2	0%
Clostridium cadaveris	2	0%
Pseudarthrobacter polychromogenes	2	0%
Psychrobacter pulmonis	2	0%
Natranaerovirga pectinivora	2	0%
unclassified Petrimonas	2	0%
Atopobium fossor	2	0%
Blautia faecis	2	0%
Enterococcus durans	2	0%
Bacteroides ovatus	2	0%
Pseudoalteromonas marina	2	0%
Staphylococcus succinus	2	0%
unclassified Paludibacter	1	0%
Aerococcus urinaeequi	1	0%
Vagococcus fessus	1	0%
Bacteroides pectinophilus	1	0%
Clostridium chauvoei	1	0%
Lactobacillus coleohominis	1	0%
Salirhabdus euzebyi	1	0%
Staphylococcus kloosii	1	0%
unclassified Pseudoalteromonas	1	0%
Stomatobaculum longum	1	0%
Pseudoalteromonas lipolytica	1	0%
unclassified Clostridium	1	0%
Agathobacter ruminis	1	0%
Lactococcus raffinolactis	1	0%
unclassified Bulleidia	1	0%
Frondihabitans australicus	1	0%
Actinomyces turicensis	1	0%
unclassified Dorea	1	0%
Bifidobacterium callitrichos	1	0%
Streptococcus thoraltensis	1	0%

Glutamicibacter ardleyensis	1	0%
Anaerofustis stercorihominis	1	0%
Oceanisphaera sediminis	1	0%
unclassified Bacillus	1	0%
unclassified Nocardioides	1	0%
Parabacteroides johnsonii	1	0%
Bacteroides gallinaceum	1	0%
Brassicibacter thermophilus	1	0%
Vibrio penaeicida	1	0%
unclassified Prevotella	1	0%
Shigella boydii	1	0%
unclassified Deltaproteobacteria	1	0%
Rhizobium loessense	1	0%
Carnobacterium pleistocenium	1	0%
Streptococcus porcorum	1	0%
Vagococcus fluvialis	1	0%
Clostridium tertium	1	0%
Clostridium leptum	1	0%
Shigella flexneri	1	0%
Pseudoalteromonas luteoviolacea	1	0%
Lactobacillus oris	1	0%
Turicibacter sanguinis	1	0%
Actinomyces meyeri	1	0%
Clostridium colicanis	1	0%
Bariatricus massiliensis	1	0%
Abyssivirga alkaniphila	1	0%
Bacteroides dorei	1	0%
Clostridium quinii	1	0%
Proteiniborus ethanoligenes	1	0%
Proteinivorax tanatarense	1	0%
Haemophilus sputorum	1	0%
unclassified Dietzia	1	0%
Lactonifactor longoviformis	1	0%
Bacillus subtilis	1	0%

Streptomyces rubrus	1	0%
Veillonella magna	1	0%
Aerococcus suis	1	0%
Rhodobacter capsulatus	1	0%
Sphingomonas faeni	1	0%
Jeotgalibaca dankookensis	1	0%
Aerococcus sanguinicola	1	0%
Pseudoclavibacter helvolus	1	0%
Lactobacillus letivazi	1	0%
Peptoclostridium litorale	1	0%
Pseudomonas borbori	1	0%
Trueperella abortisuis	1	0%
Shuttleworthia satelles	1	0%
Paeniglutamicibacter antarcticus	1	0%
Pseudomonas protegens	1	0%
Streptococcus caprae	1	0%
Renibacterium salmoninarum	1	0%
Blautia coccoides	1	0%
Pseudarthrobacter sulfonivorans	1	0%
Pseudoalteromonas flavipulchra	1	0%
Pseudoalteromonas haloplanktis	1	0%
Bacteroides cellulosilyticus	1	0%
Leucobacter zeae	1	0%
Prevotella buccalis	1	0%
Psychrobacter alimentarius	1	0%
Bifidobacterium indicum	1	0%
Fusobacterium periodonticum	1	0%
Lactobacillus saniviri	1	0%
unclassified Enterococcus	1	0%
Eubacterium rectale	1	0%
Metabacterium polyspora	1	0%
Actinomyces odontolyticus	1	0%
Bacillus cereus	1	0%
Sphingobacterium anhuiense	1	0%

Cutibacterium acnes	1	0%
Oscillospira guilliermondii	1	0%
Bacillus tequilensis	1	0%
Leuconostoc gelidum	1	0%
Microbacterium lacticum	1	0%
Corynebacterium urealyticum	1	0%
Pediococcus ethanolidurans	1	0%
unclassified Sphingobacterium	1	0%
Ruminococcus lactaris	1	0%
Limnobacter thiooxidans	1	0%
unclassified Alphaproteobacteria	1	0%
Alloprevotella tannerae	1	0%
Neptuniibacter caesariensis	1	0%
Corynebacterium stationis	1	0%
Carnobacterium inhibens	1	0%
Enterococcus casseliflavus	1	0%
Clostridium phoceensis	1	0%
Clostridium botulinum	1	0%
Intestinimonas timonensis	1	0%
Enterobacter hormaechei	1	0%
Oscillibacter ruminantium	1	0%
Clostridium bovipellis	1	0%
Vagococcus carniphilus	1	0%
Pseudoalteromonas nigrifaciens	1	0%
unclassified Lachnospiraceae	1	0%
Streptococcus thermophilus	1	0%
Bifidobacterium bifidum	1	0%
Corynebacterium argentoratense	1	0%
Arthrobacter cryoconiti	1	0%
Dietzia lutea	1	0%
Lactobacillus perolens	1	0%
Lactobacillus malefermentans	1	0%
Veillonella parvula	1	0%
Erysipelothrix rhusiopathiae	1	0%

Acidaminococcus intestini	1	0%
Anaerocolumna jejuensis	1	0%
Alistipes indistinctus	1	0%
Glutamicibacter bergerei	1	0%
Lachnoanaerobaculum cf.	1	0%
Blautia wexlerae	1	0%
Streptomyces flavogriseus	1	0%
Streptococcus equi	1	0%
Zhihengliuella alba	1	0%
Bifidobacterium pseudolongum	1	0%
Catabacter hongkongensis	1	0%
Bacteroides rodentium	1	0%
Actinomyces radicidentis	1	0%
Clostridium cocleatum	1	0%
Ruminiclostridium thermocellum	1	0%
unclassified Clostridiales	1	0%
Clostridium sartagoforme	1	0%
Brevundimonas nasdae	1	0%
Moraxella nonliquefaciens	1	0%
Lactobacillus casei	1	0%
Christensenella minuta	1	0%
Pediococcus claussenii	1	0%
Pseudoalteromonas tetraodonis	1	0%
Pseudoalteromonas citrea	1	0%
Izhakiella capsodis	1	0%
Rhodococcus erythropolis	1	0%
Arthrobacter agilis	1	0%
Shewanella chilikensis	1	0%
Vibrio litoralis	1	0%
Serratia liquefaciens	1	0%
Luteococcus sanguinis	1	0%
Eubacterium dolichum	1	0%
Neorhizobium huautlense	1	0%
Clostridium cellulolyticum	1	0%

Vallitalea pronyensis	1	0%
Bifidobacterium tissieri	1	0%
Methylobacterium brachiatum	1	0%
Clostridium indolis	1	0%
Ethanoligenens harbinense	1	0%
Clostridium lavalense	1	0%
Actinobacillus rossii	1	0%
Clostridium aerotolerans	1	0%
Enterococcus mundtii	1	0%
Pseudobutyrivibrio ruminis	1	0%
Clostridium celatum	1	0%
Vibrio alginolyticus	1	0%
Dorea longicatena	1	0%

Table S7. Bacterial species in the feces of calves fed with milk replacer only (CON group) after 14 days of the experiment.

Bacterial species in CON group after experiment	Number of reads	Relative abundance
Ruminococcus torques	6879	25.5%
Blautia wexlerae	3197	11.85%
Butyricicoccus pullicaecorum	2864	10.61%
Tyzzerella nexilis	2738	10.15%
Bacteroides vulgatus	1003	3.71%
Clostridium spiroforme	963	3.57%
Ruminococcus faecis	961	3.56%
Bacteroides coprophilus	871	3.22%
Faecalibacterium prausnitzii	835	3.09%
Clostridium lactatifermentans	703	2.6%
Lactobacillus reuteri	514	1.9%
Bifidobacterium pseudocatenulatum	430	1.59%
Bacteroides thetaiotaomicron	406	1.5%
Blautia obeum	399	1.47%
Bacteroides salanitronis	371	1.37%
Escherichia coli	306	1.13%
Bacteroides helcogenes	262	0.97%

Prevotella copri	259	0.96%
Ruminococcus gnavus	218	0.8%
Blautia massiliensis	200	0.74%
Unclassified	181	0.67%
Bifidobacterium longum	143	0.53%
Eubacterium fissicatena	127	0.47%
Bacteroides uniformis	118	0.43%
Dorea formicigenerans	113	0.41%
Coprococcus comes	104	0.38%
Howardella ureilytica	97	0.35%
Bacteroides coprocola	88	0.32%
Hespellia porcina	64	0.23%
Clostridium fusiformis	61	0.22%
Holdemanella biformis	58	0.21%
Bifidobacterium scardovii	55	0.2%
Lactobacillus johnsonii	52	0.19%
Bifidobacterium catenulatum	46	0.17%
Eubacterium contortum	42	0.15%
Murimonas intestini	40	0.14%
Ruminococcus bromii	36	0.13%
Streptococcus thermophilus	35	0.12%
Anaerostipes caccae	34	0.12%
Eubacterium oxidoreducens	30	0.11%
Alloprevotella rava	30	0.11%
Kluyvera georgiana	29	0.1%
Blautia luti	28	0.1%
Streptococcus lutetiensis	27	0.1%
unclassified Bacteroidaceae	25	0.09%
Bacteroides plebeius	25	0.09%
Streptococcus gallolyticus	24	0.08%
Actinomyces hyovaginalis	24	0.08%
Lactobacillus mucosae	23	0.08%
Actinomyces cardiffensis	19	0.07%
Butyrivibrio fibrisolvens	19	0.07%

Bacteroides acidifaciens	19	0.07%
Blautia hydrogenotrophica	18	0.06%
unclassified Clostridium	18	0.06%
Corynebacterium provencense	17	0.06%
unclassified Coprobacillus	16	0.05%
Mobilitalea sibirica	16	0.05%
Pseudobutyrivibrio xylanivorans	16	0.05%
Blautia producta	16	0.05%
Bacteroides ovatus	16	0.05%
Bacteroides faecis	16	0.05%
Prevotella histicola	13	0.04%
Eubacterium ruminantium	13	0.04%
Clostridium polysaccharolyticum	13	0.04%
Bifidobacterium kashiwanohense	12	0.04%
Clostridium saccharolyticum	12	0.04%
Casaltella massiliensis	12	0.04%
Lactobacillus amylovorus	11	0.04%
Clostridium indolis	11	0.04%
Bacteroides dorei	10	0.03%
Shigella dysenteriae	10	0.03%
Faecalicoccus pleomorphus	10	0.03%
Blautia glucerasea	10	0.03%
Bifidobacterium pullorum	10	0.03%
unclassified Lachnospiraceae	9	0.03%
Clostridium oroticum	9	0.03%
Bacteroides faecichinchillae	9	0.03%
Blautia schinkii	9	0.03%
Sutterella stercoricanis	8	0.02%
unclassified Bacteroidales	8	0.02%
Ruminococcus gauvreauii	8	0.02%
Pseudoflavonifractor capillosus	8	0.02%
Bifidobacterium breve	8	0.02%
Roseburia faecis	8	0.02%
Lactobacillus panis	7	0.02%

Clostridium aldenense	7	0.02%
Actinomyces nasicola	7	0.02%
Anaerococcus provenciensis	7	0.02%
Streptococcus equinus	7	0.02%
Clostridium glycyrrhizinilyticum	7	0.02%
Candidatus Dorea	7	0.02%
Lactonifactor longoviformis	7	0.02%
Ewingella americana	6	0.02%
Clostridium populeti	6	0.02%
Bacteroides barnesiae	6	0.02%
unclassified Bifidobacterium	6	0.02%
Lactobacillus antri	6	0.02%
Roseburia hominis	6	0.02%
unclassified Lactobacillus	6	0.02%
Bacteroides stercoris	6	0.02%
Parabacteroides johnsonii	5	0.01%
Ruminococcus lactaris	5	0.01%
Kosakonia sacchari	5	0.01%
Eisenbergiella tayi	5	0.01%
Flavonifractor plautii	5	0.01%
Corynebacterium urealyticum	5	0.01%
Clostridium neopropionicum	5	0.01%
Lactococcus lactis	5	0.01%
Bifidobacterium stellenboschense	5	0.01%
Gemmiger formicilis	5	0.01%
Clostridium viride	4	0.01%
Peptostreptococcus russellii	4	0.01%
Anaerococcus prevotii	4	0.01%
Clostridium cocleatum	4	0.01%
Sharpea azabuensis	4	0.01%
Escherichia albertii	4	0.01%
Pseudobutyrivibrio ruminis	4	0.01%
Hungatella hathewayi	4	0.01%
Stomatobaculum longum	4	0.01%

Bifidobacterium tissieri	4	0.01%
Lactobacillus frumenti	4	0.01%
unclassified Bacteroides	3	0.01%
Coprococcus eutactus	3	0.01%
Catenibacterium mitsuokai	3	0.01%
Anaerococcus tetradius	3	0.01%
Actinomyces odontolyticus	3	0.01%
Clostridium clostridioforme	3	0.01%
Bacteroides fragilis	3	0.01%
Bifidobacterium indicum	3	0.01%
Flintibacter butyricus	3	0.01%
Clostridium sphenoides	3	0.01%
Bacteroides paurosaccharolyticus	3	0.01%
Anaerostipes butyraticus	3	0.01%
Bifidobacterium biavatii	3	0.01%
Blautia stercoris	3	0.01%
Clostridium innocuum	3	0.01%
Fournierella massiliensis	3	0.01%
Erysipelatoclostridium ramosum	3	0.01%
Bacteroides cellulosilyticus	2	0%
Lachnospiraceae oral	2	0%
Bifidobacterium gallicum	2	0%
Bacteroides massiliensis	2	0%
Clostridium paraputrificum	2	0%
Phascolarctobacterium succinatutens	2	0%
Parvimonas micra	2	0%
unclassified Ruminococcus	2	0%
Lactobacillus gasseri	2	0%
Photorhabdus luminescens	2	0%
Serratia marcescens	2	0%
unclassified Prevotella	2	0%
Staphylococcus epidermidis	2	0%
Anaerocolumna xylanovorans	2	0%
Clostridium cellulolyticum	2	0%

unclassified Wautersiella	2	0%
Clostridium hylemonae	2	0%
Oribacterium sinus	2	0%
Anaerocolumna aminovalerica	2	0%
Bacteroides oleiciplenus	2	0%
Comamonas kerstersii	2	0%
Clostridium perfringens	2	0%
Prevotella genomosp.	2	0%
Eubacterium minutum	2	0%
Anaerobium acetethylicum	2	0%
Clostridium celerecrescens	2	0%
Robinsoniella peoriensis	2	0%
Clostridium saccharogumia	1	0%
Lactobacillus delbrueckii	1	0%
Lactobacillus salivarius	1	0%
Peptostreptococcus anaerobius	1	0%
Coprococcus catus	1	0%
Bacteroides finegoldii	1	0%
Lactobacillus coleohominis	1	0%
Turicibacter sanguinis	1	0%
Gallibacterium anatis	1	0%
Coprobacillus cateniformis	1	0%
unclassified Peptostreptococcaceae	1	0%
Mogibacterium diversum	1	0%
Intestinimonas gabonensis	1	0%
Clostridium scindens	1	0%
Enterobacter hormaechei	1	0%
Citrobacter koseri	1	0%
Fusicatenibacter saccharivorans	1	0%
Bacteroides sartorii	1	0%
Anaerostipes rhamnosivorans	1	0%
Cutibacterium acnes	1	0%
Peptostreptococcus stomatis	1	0%
Blautia coccoides	1	0%

Aquicella siphonis	1	0%
Clostridium amygdalinum	1	0%
Klebsiella oxytoca	1	0%
Clostridium methylpentosum	1	0%
Eubacterium hallii	1	0%
unclassified Acetivibrio	1	0%
Bifidobacterium saguini	1	0%
Bacteroides intestinalis	1	0%
unclassified Clostridiales	1	0%
Lactobacillus rhamnosus	1	0%
Anaerotaenia torta	1	0%
unclassified Lactobacillaceae	1	0%
Bacteroides fluxus	1	0%
Bifidobacterium coryneforme	1	0%
Enterococcus casseliflavus	1	0%
unclassified Gammaproteobacteria	1	0%
Lactobacillus tucceti	1	0%
Anaerobacterium chartisolvens	1	0%
Roseburia inulinivorans	1	0%
Salmonella enterica	1	0%
unclassified Pseudobutyrivibrio	1	0%
Bacteroides zoogleoformans	1	0%
Corynebacterium amycolatum	1	0%
Drancourtella massiliensis	1	0%
Natranaerovirga pectinivora	1	0%
Lactobacillus fermentum	1	0%
Afipia broomeae	1	0%
Eubacterium desmolans	1	0%
Flavobacterium johnsoniae	1	0%
unclassified Johnsonella	1	0%
Desulfotomaculum guttoideum	1	0%
Lachnospira pectinoschiza	1	0%
Ralstonia pickettii	1	0%
Clostridium quinii	1	0%

Bifidobacterium angulatum	1	0%
unclassified Eubacterium	1	0%
Acetobacter sicerae	1	0%
Achromobacter marplatensis	1	0%
Bacteroides caccae	1	0%
Bacteroides pectinophilus	1	0%
Eubacterium cellulosolvens	1	0%
Bifidobacterium saeculare	1	0%
Gottschalkia acidurici	1	0%
unclassified Erysipelotrichaceae	1	0%
Pantoea eucrina	1	0%
Falcatimonas natans	1	0%
Lachnoanaerobaculum saburreum	1	0%
unclassified Prevotellaceae	1	0%
Hespellia stercorisuis	1	0%
Bariatricus massiliensis	1	0%
Streptococcus parasanguinis	1	0%
Sulfitobacter mediterraneus	1	0%
Shigella flexneri	1	0%
Lautropia mirabilis	1	0%
Dysgonomonas termitidis	1	0%
Eubacterium dolichum	1	0%
Crassaminicella profunda	1	0%
unclassified Clostridia	1	0%
Clostridium asparagiforme	1	0%
Streptococcus danieliae	1	0%
Bacteroides caecigallinarum	1	0%
Bilophila wadsworthia	1	0%
Bacteroides xylanisolvens	1	0%
Peptococcus niger	1	0%
Peptoniphilus tyrrelliae	1	0%
Subdoligranulum variabile	1	0%
Vallitalea pronyensis	1	0%
Bacillus horneckiae	1	0%

Table S8. Volatile compounds (%) profile of calves feces in control (CON) and supplemented with fermented milk permeate (MP) groups.

		CON0		CON14		MP0		MP14	
**RT**	**Analyte**	**average**	**stdev**	**average**	**stdev**	**average**	**stdev**	**average**	**stdev**
2.355	Acetic acid	3.7061	0.31	2.24447	0.18	1.9507	0.11	0.6407	0.05
3.815	Acetoin	7.3214	0.62	0	0	1.3966	0.25	0	0
4.082	Propanoic acid	0	0	2.62306	0.18	0	0	2.1949	0.14
4.338	1-Butanol. 3-methyl-	0	0	0	0	2.9003	0.17	0	0
4.393	1-Pentanol	0.6837	0.04	0	0	0	0	0	0
5.61	2.3-Butanediol	0.37	0.02	0	0	0.3148	0.05	0	0
6.338	Butanoic acid	9.5251	0.59	32.3882	2.84	22.134	1.93	24.938	2.1
7.172	Butanoic acid. 3-methyl-	2.3571	0.24	2.33326	0.18	0.5918	0.04	2.2063	0.22
7.277	Butyric acid <2-methyl->	4.2434	0.38	4.27478	0.29	1.0899	0.09	2.6514	0.17
8.155	2-Hexanone. 4-methyl-	0	0	0	0	0.0607	0.01	0	0
8.176	2-Heptanone	6.7662	0.34	0	0	2.5394	0.24	0	0
8.188	Pentanoic acid	0.219	0.018	5.87319	0.41	0.1711	0.01	2.4026	0.15
8.388	Butanoic acid. propyl ester	0	0	0	0	0.4715	0.03	0	0
8.411	Butanoate <propyl->	0	0	0	0	0	0	0.3399	0.02
8.692	Oxime-. methoxy-phenyl-_	2.0654	0.21	0	0	1.504	0.14	0	0
9.06	Dimethyl sulfone	0	0	0	0	0.0177	0.002	0	0
9.508	Ethyl tiglate	0.1486	0.005	0	0	0	0	0	0
9.742	Hexanoic acid. 2-methyl-	0	0	0.02408	0.003	0	0	0	0
9.771	Butanoic acid. 3-methyl-. propyl ester	0.067	0.004	0	0	0	0	0	0
9.883	Pentanoic acid. 4-methyl-	0.1096	0.02	0.09882	0.01	0	0	0.1556	0.02
9.999	Benzaldehyde	0	0	0.38298	0.03	0.3607	0.04	0.0338	0.001
10.111	2-Heptanone. 5-methyl-	0	0	0.12479	0.01	0.028	0.002	0.0358	0.003
10.275	Dimethyl trisulfide	0	0	0	0	0	0	1.2237	0.14
10.515	Hexanoic acid	2.5125	0.18	1.45552	0.11	3.8874	0.28	1.615	0.11
10.525	Vinyl amyl carbinol	0	0	0.00499	0.001	0	0	0	0
10.602	Phosphonic acid. (p-hydroxyphenyl)-	0	0	0.47788	0.04	0	0	3.7842	0.03
10.61	Phenol	0	0	1.17612	0.15	0.1559	0.03	0.9699	0.06
10.717	3(2H)-Thiophenone. dihydro-2-methyl-	0	0	0.03791	0.005	0	0	0	0

10.81	2-Octanone	1.3566	0.12	0	0	0.8887	0.07	0	0
10.9	Benzene. 1.2.4-trimethyl-	0	0	0.02967	0.005	0	0	0.0237	0.006
10.925	Mesitylene	0	0	0	0	0	0	0.079	0.001
10.937	Butanoic acid. butyl ester	0.0544	0.002	0	0	0	0	0	0
10.949	Butanoate <butyl->	0	0	0.03683	0.003	0	0	0.0853	0.002
10.955	Propyl valerate	0	0	0.06729	0.003	0	0	0	0
11.605	2.4-Heptanedione. 6-methyl-	0	0	0.0255	0.002	0	0	0	0
11.775	1-Hexanol. 2-ethyl-	5.9954	0.35	1.71866	0.21	1.491	0.15	3.0568	0.28
11.806	2-Propyl-1-pentanol	0.0105	0.001	0	0	9.6066	0.46	0	0
11.915	Benzyl alcohol	0	0	0.19703	0.02	0.0618	0.01	0.0753	0.02
12.151	Phenylacetaldehyde	0	0	0.02488	0.003	0	0	0	0
12.17	Benzeneacetaldehyde	0.3557	0.03	0.02563	0.005	0.0456	0.002	0	0
12.221	Butanoic acid. 3-methyl-. butyl ester	0	0	0	0	0	0	0.0343	0.005
12.355	Benzene. 1-methyl-4-propyl-	0	0	0	0	0	0	0.0172	0.002
12.495	Octen-1-al<2E->	0	0	0	0	0	0	0.0622	0.003
12.504	Propanoic acid. 2-methyl-. 2-methylbutyl ester	0	0	0.05093	0.003	0	0	0	0
12.514	2-Octenal. (E)-	0	0	0.01841	0.002	0	0	0.0487	0.004
12.514	Butanoic acid. 3-methylbutyl ester	0.07	0.005	0	0	0	0	0	0
12.524	Heptane. 3.3.5-trimethyl-	0	0	0.02892	0.003	0	0	0	0
12.545	Butanoate. <2-Methylbutyl ->	0	0	0	0	0	0	0.0327	0.004
12.77	Cyclooctyl alcohol	0	0	0	0	0	0	0.0313	0.005
12.78	Benzene. (1-methylpropyl)-	0	0	0.00546	0.0001	0	0	0	0
12.815	1-Octanol	0.0235	0.003	0.01296	0.0317	0	0	0.1986	0.02
12.815	Acetophenone	0	0	0	0	0	0	0.0192	0.002
12.826	Chloromethyl octyl ether	0	0	0	0	0.0087	0.001	0	0
12.831	Formic acid. octyl ester	0	0	0.01908	0.002	0	0	0	0
12.872	Heptanoic acid	0.6885	0.06	0	0	0.2161	0.02	0	0
12.963	p-Cresol	0.9924	0.07	21.984	0.93	1.5253	0.17	26.559	1.48
13.01	Benzene. 2-ethyl-1.4-dimethyl-	0	0	0.01457	0.002	0	0	0.151	0.02
13.025	Phenol. 3-methyl-	0	0	0	0	0	0	0.0342	0.001
13.12	4-Hexen-1-ol. (4E)-. acetate	0.1329	0.06	0	0	0	0	0	0
13.123	8-Nonen-2-one	0	0	0	0	0.0156	0.001	0	0

13.125	7-Octen-2-one. 6-methyl-	0.1168	0.003	0	0	0	0	0	0
13.234	Benzene. 1-ethyl-2.4-dimethyl-	0	0	0.03352	0.004	0	0	0.0489	0.005
13.345	2-Nonanone	9.6406	0.74	0.20703	0.021	6.4706	0.43	0.1729	0.021
13.399	Chimilether	0	0	0	0	0	0	0.0379	0.004
13.408	Hexanoate <propyl->	0	0	0.05593	0.006	0.2324	0.02	0.1318	0.01
13.534	Methoxyacetic acid. 3-tridecyl ester	0	0	0	0	0	0	0.0631	0.01
13.542	Methoxyacetic acid. 4-tetradecyl ester	0	0	0.02975	0.003	0	0	0	0
13.543	Undecane <n->	0.5628	0.042	0.04985	0.004	0.0788	0.003	0.0255	0.002
13.544	Methoxyacetic acid. 3-tetradecyl ester	0	0	0.0121	0.002	0	0	0.0237	0.004
13.553	Methoxyacetic acid. 2-tridecyl ester	0	0	0.01666	0.003	0	0	0	0
13.573	Methoxyacetic acid. 6-ethyl-3-octyl ester	0	0	0	0	0	0	0.012	0.004
13.577	2-Nonanol	0	0	0.00743	0.001	0.0524	0.003	0.0176	0.001
13.638	Nonanal	0.1346	0.013	0.28662	0.018	0.2592	0.02	0.2319	0.03
13.689	Ethanone. 1-(4.5-dihydro-2-thiazolyl)-	0	0	0.06576	0.01	0	0	0	0
13.745	Butanoic acid. 3-methyl-. 3-methylbutyl ester	0	0	0	0	0	0	0.0104	0.003
13.811	Cyclohexanol. 2.6-dimethyl-	0.1679	0.021	0	0	0.0945	0.0015	0	0
13.897	Phenethyl alcohol	1.9454	0.16	0.43642	0.05	2.3464	0.19	0.6267	0.05
13.985	Benzoic acid <hexahydro->	0	0	0.00903	0.001	0	0	0	0
14.097	Benzene. 1-ethyl-3.5-dimethyl-	0	0	0.09364	0.003	0	0	0.0965	0.004
14.106	Octanoic acid. methyl ester	0	0	0	0	0	0	0.0102	0.002
14.279	Benzyl methyl ketone	0	0	0	0	0	0	0.0091	0.001
14.343	1-Hexanol. 4-methyl-. (S)-	0	0	0	0	0.0108	0.003	0	0
14.56	Indan. 1-methyl-	0	0	0.04174	0.0021	0	0	0	0
14.727	Acetate <hexyl-. 2-ethyl->	0.0267	0.003	0.04336	0.002	0.0067	0.001	0	0
14.896	Methyl octyl ketone	0.4488	0.015	0.17899	0.012	0.0297	0.005	0.208	0.011
14.948	2-Nonenal. (E)-	0	0	0.04692	0.004	0.0286	0.003	0.028	0.002
15.035	Nonane. 5-(2-methylpropyl)-	0	0	0	0	0.012	0.002	0	0
15.091	Naphthalene. 1.2.3.4-tetrahydro-	0	0	0.12138	0.003	0	0	0.091	0.002
15.17	Phenol. 2-ethyl-	0	0	0	0	0	0	0.0137	0.003
15.204	Octanoic acid	7.4339	0.51	0.52065	0.04	5.9875	0.47	0.7271	0.06

15.221	6.6-Dimethyl-4.5-tetramethylene-2-phenyl-5.6-dihydro-4H-1.3-oxazine	0	0	0.02699	0.003	0	0	0	0
15.23	Nonanol	0	0	0.02245	0.003	0	0	0	0
15.329	2-Piperidinone	0.0263	0.003	0.96085	0.08	0.2107	0.02	1.1545	0.12
15.499	Undeca-1.3.5-triene	0.0924	0.008	0	0	0	0	0	0
15.693	1H-Indene. 2.3-dihydro-1.6-dimethyl-	0	0	0.02945	0.003	0	0	0	0
15.701	2-Decanone	0	0	0.08568	0.009	0	0	0.0178	0.002
15.785	Octanoate <ethyl->	0.2284	0.021	0.08985	0.009	0.2825	0.023	1.0224	0.14
15.859	Dodecane	1.2208	0.18	0.14616	0.013	0.8224	0.07	0.1582	0.012
15.868	Nonane. 2-methyl-5-propyl-	0	0	0.00773	0.001	0	0	0	0
15.965	Naphthalene. 1.2.3.4-tetrahydro-1-methyl-	0	0	0.01302	0.002	0	0	0	0
16.006	Decanal	0	0	0.02937	0.0021	0.0178	0.0011	0.0416	0.004
16.16	Benzene. 1-ethyl-2.4.5-trimethyl-	0	0	0	0	0	0	0.0091	1E-04
16.178	Sulfurous acid. dodecyl 2-ethylhexyl ester	0	0	0	0	0.0065	0.0004	0	0
16.342	Naphthalene. 1.2.3.4-tetrahydro-2-methyl-	0	0	0.08243	0.0023	0	0	0.0698	0.001
16.395	Tetrasulfide <methyl->	0	0	0	0	0	0	0.8258	0.025
16.443	1-Cyclohexene-1-carboxaldehyde. 2.6.6-trimethyl-	0.1073	0.015	0.05506	0.004	0.0074	0.0004	0.0182	0.002
16.452	Cyclocitral<beta->	0	0	0	0	0.0171	0.002	0	0
16.561	Benzothiazole	0	0	0.00771	0.0001	0.0163	0.002	0.0289	0.003
16.565	1.2-Benzisothiazole	0	0	0	0	0	0	0.0085	0.001
16.616	Phenol. 2-amino-4-methoxy-	0	0	0	0	0.1034	0.011	0	0
16.749	Trichloroacetic acid. 2-ethylhexyl ester	0	0	0.03579	0.003	0	0	0	0
16.794	3.4-Dimethylcyclohexanol	0	0	0	0	0.022	0.003	0	0
16.83	Quinoline	0	0	0.01208	0.0023	0	0	0	0
16.833	Benzeneacetonitrile. .alpha.-methylene-	0	0	0.00531	0.0001	0	0	0	0
16.906	Benzeneacetic acid. ethyl ester	0.0284	0.003	0	0	0	0	0.0057	0.001
17.043	Ethosuximide	0	0	0.0109	0.003	0.0156	0.002	0.0037	3E-04
17.099	Benzene. 1-methyl-3-(1-methyl-2-propenyl)-	0	0	0.02158	0.004	0	0	0	0

17.101	2.2-Dimethylindene. 2.3-dihydro-	0	0	0.00999	0.001	0	0	0	0
17.161	Acetic acid. 2-phenylethyl ester	0.0305	0.0023	0	0	0.045	0.0025	0	0
17.177	Phenethyl isobutyrate	0.0141	0.002	0	0	0	0	0	0
17.25	Dec-(2E)-enal	0	0	0.00614	0.0001	0.0067	0.002	0.0048	0.001
17.354	Nonanoic acid	12.172	1.23	1.12503	0.56	10.394	1.23	0.921	0.11
17.365	Phenol. 3-[(trimethylsilyl)oxy]-	0	0	0	0	0	0	0.0166	0.005
17.439	Naphthalene. 1.2.3.4-tetrahydro-5-methyl-	0	0	0.05576	0.0884	0.0173	0.0548	0.1771	0.434
17.458	1-Decanol	0.0258	0.003	0	0	0.0633	0.005	0	0
17.458	Naphthalene. 1.2.3.4-tetrahydro-6-methyl-	0	0	0.19697	0.021	0	0	0.1139	0.012
17.562	Phenol. 3-methyl-4-(methylthio)-	0	0	0	0	0	0	0.0169	0.002
17.649	Dodecane. 4.6-dimethyl-	0	0	0.0305	0.003	0	0	0	0
17.865	Propyl octanoate	0.006	0.001	0.04706	0.003	0.0935	0.005	0.2081	0.001
17.903	Octanoic acid. octyl ester	0.0568	0.004	0	0	0	0	0	0
17.917	2-Undecanone	0.2119	0.18	0.2206	0.015	0.1581	0.011	0.206	0.019
17.947	Nonanoate <ethyl->	0.0821	0.004	0.10129	0.015	0.0768	0.0032	0.241	0.018
18.023	Indole	6.8668	0.65	11.9005	0.92	12.24	0.87	12.156	1.18
18.206	Undecanal	0	0	0.0155	0.009	0.0041	0.0003	0.0047	1E-04
18.213	1-Decanol. 2-hexyl-	0	0	0.00701	0.001	0	0	0	0
18.535	n-Butyric acid 2-ethylhexyl ester	0.0304	0.0045	0.14576	0.031	0	0	0	0
18.695	Naphthalene. 1.2.3.4-tetrahydro-1.5-dimethyl-	0	0	0.00957	0.0003	0	0	0	0
18.754	Benzene. 1-(1.1-dimethylethyl)-4-ethenyl-	0	0	0.01807	0.0023	0	0	0.0217	0.004
19.094	Naphthalene. 1.2.3.4-tetrahydro-2.6-dimethyl-	0	0	0.01253	0.0054	0	0	0	0
19.136	Hydrocinnamate <ethyl->	0	0	0.00561	0.001	0.0084	0.001	0	0
19.246	Decyl methyl ketone	0	0	0.0489	0.003	0	0	0.0277	0.002
19.254	9-Decenoic acid	0	0	0	0	0	0	0.0073	0.001
19.263	2-Decanone. 5.9-dimethyl-	0	0	0.00425	0.0004	0.0067	0.0005	0	0
19.271	Propanoic acid. 2-methyl-. 2.2-dimethyl-1-(2-hydroxy-1-methylethyl)propyl ester	0	0	0	0	0.0126	0.0025	0	0
19.335	n-Decanoic acid	3.095	0.32	0.66321	0.05	2.976	0.28	0.6392	0.04

19.392	Tetracosanoic acid	0	0	0	0	0	0	0.0099	0.001
19.395	2-Nonadecanone	0	0	0.06127	0.0056	0	0	0.1213	0.018
19.511	Pentanoic acid. 2-ethylhexyl ester	0	0	0.00325	0.0005	0	0	0	0
19.531	Dodecane. 1-methoxy-	0	0	0.01683	0.005	0	0	0.0242	0.003
19.621	Dodecane. 2.6.10-trimethyl-	0	0	0.01088	0.0025	0	0	0.0069	1E-04
19.823	Ethyl 9-decenoate	0	0	0	0	0.0056	0.001	0	0
19.829	Ethyl trans-4-decenoate	0	0	0.00704	0.001	0	0	0.0472	0.002
19.907	Nonanoic acid. propyl ester	0.0162	0.003	0.03257	0.005	0.0207	0.0031	0.0833	0.007
19.909	1-Tetradecyl acetate	0	0	0.01316	0.002	0	0	0	0
19.916	Nonanoic acid. decyl ester	0	0	0	0	0	0	0.0042	0.001
19.979	2-Dodecanone	0	0	0.04567	0.0023	0	0	0	0
19.989	Decanoate <ethyl->	0.8111	0.023	0.21868	0.018	0.4807	0.031	1.429	0.14
20.022	1H-Indole. 3-methyl-	0	0	0	0	0	0	0.0385	0.004
20.069	Tetradecane	0.5187	0.025	0.12994	0.013	0.3931	0.028	0.1109	0.01
20.266	Dodecanal	0.0113	0.002	0.1957	0.011	0.0098	0.0001	0.1041	0.013
20.421	2.4.7.9-Tetramethyl-5-decyn-4.7-diol	0	0	0.01889	0.002	0.0503	0.004	0.0064	1E-04
20.786	Caryophyllene	0	0	0	0	0.0213	0.0015	0	0
20.977	.beta.-Phenylethyl butyrate	0.008	0.001	0	0	0.0081	0.002	0	0
20.98	Benzyl carbyl butyrate	0	0	0	0	0	0	0.002	1E-04
21.139	5.9-Undecadien-2-one. 6.10-dimethyl-. (Z)-	0	0	0	0	0	0	0.0242	0.003
21.243	2-Tridecanone	0	0	0	0	0	0	0	0
21.282	Triacontane	0	0	0.01487	0.0013	0	0	0	0
21.284	Pentadecane. 2.6.10.14-tetramethyl-	0	0	0.01178	0.002	0	0	0.0101	0.003
21.298	Docosane <n->	0	0	0.00901	0.001	0	0	0.007	0.002
21.37	Supraene	0	0	0.02208	0.0018	0	0	0	0
21.374	7.11-Dimethyldodeca-2.6.10-trien-1-ol	0	0	0.0127	0.003	0	0	0	0
21.382	3.7.11-Tridecatrienenitrile. 4.8.12-trimethyl-	0	0	0.02134	0.0016	0	0	0.0299	0.001
21.542	2.5-Cyclohexadiene-1.4-dione. 2.6-bis(1.1-dimethylethyl)-	0	0	0.06671	0.003	0	0	0.2063	0.015
21.579	trans-2-Dodecen-1-ol. trifluoroacetate	0	0	0	0	0	0	0.0123	0.003

21.68	Butyl 9-decenoate	0	0	0.00512	0.0001	0	0	0.0074	0.001
21.816	Decanoic acid. propyl ester	0.1079	0.016	0.07896	0.011	0.0702	0.009	0.2366	0.018
21.9	3-Buten-2-one. 4-(2.6.6-trimethyl-1-cyclohexen-1-yl)-	0.0976	0.006	0	0	0.0319	0.004	0	0
21.904	Undecanoic acid. ethyl ester	0	0	0	0	0	0	0.0106	0.003
21.916	Trifluoroacetic acid.n-tridecyl ester	0	0	0	0	0	0	0.027	0.002
21.99	Nonane. 3-methyl-5-propyl-	0	0	0	0	0	0	0.028	0.003
21.99	Pentadecane <n->	0.2059	0.015	0.03954	0.0034	0	0	0.0368	0.004
22.06	Methyl nonyl carbinol	0	0	0.00131	0.0001	0	0	0	0
22.233	Tridecanal	0	0	0.01685	0.0023	0	0	0.0072	0.002
22.374	Butylated Hydroxytoluene	0	0	0.01436	0.002	0	0	0.0266	0.003
22.514	(-)-1-Methylbutyl decanoate	0.0211	0.0021	0	0	0	0	0	0
22.837	Ethanone. 1-(3.4-dimethoxyphenyl)-	0	0	0.00434	0.0001	0	0	0	0
22.864	2(4H)-Benzofuranone. 5.6.7.7a-tetrahydro-4.4.7a-trimethyl-. (R)-	0	0	0	0	0	0	0.0032	2E-04
23.095	Dodecanoic acid	0.6084	0.039	0.31823	0.034	0.5502	0.041	0.2264	0.032
23.29	2-Tetradecanone	0	0	0.03184	0.0029	0	0	0.038	0.002
23.454	n-Tridecan-1-ol	0	0	0	0	0	0	0.0302	0.002
23.584	Butyl caprate	0	0	0	0	0	0	0.006	5E-04
23.692	Docosanoic acid. ethyl ester	0	0	0.02143	0.003	0	0	0	0
23.696	1-Nonadecene	0	0	0	0	0	0	0.0096	3E-04
23.701	Dodecanoate <ethyl->	0.4201	0.023	0.21617	0.017	0.21	0.021	0.8149	0.065
23.818	Heneicosane	0.0145	0.002	0.12848	0.015	0.0465	0.006	0.0652	0.008
23.88	Propanoic acid. 2-methyl-. 1-(1.1-dimethylethyl)-2-methyl-1.3-propanediyl ester	0.1016	0.011	0.01775	0.006	0.0221	0.003	0.0183	0.002
23.9	Sulfurous acid. octadecyl 2-pentyl ester	0	0	0	0	0	0	0.0019	2E-04
23.9	Pentanoic acid. 2.2.4-trimethyl-3-carboxyisopropyl. isobutyl ester	0	0	0.01681	0.0025	0	0	0	0
24.06	Tetradecanal	0.057	0.0041	0.21441	0.018	0.0289	0.0025	0.1016	0.018
25.055	cis-9-Tetradecen-1-ol	0	0	0.00929	0.0001	0	0	0	0
25.135	Z-8-Tetradecen-1-yl acetate	0	0	0	0	0	0	0.0059	5E-04
25.171	Heptadecanol <n->	0	0	0.03803	0.014	0	0	0.0148	0.002

25.194	Oleyl alcohol. trifluoroacetate	0	0	0.0046	0.0003	0	0	0	0
25.195	Pentadecanal-	0.01	0.0038	0	0	0	0	0	0
25.202	trans-2-Dodecen-1-ol. heptafluorobutyrate	0	0	0	0	0	0	0.0217	0.001
25.229	1-Tetradecanol	0	0	0	0	0	0	0.0415	0.002
25.307	10-Methyldodecan-4-olide	0	0	0.04751	0.0015	0	0	0.03	0.002
25.33	Dodecalactone <gamma->	0	0	0.00598	0.0001	0	0	0.0123	0.002
25.381	Dodecanoic acid. propyl ester	0.0142	0.002	0.03639	0.0015	0.0172	0.0036	0.0947	0.004
25.528	Dodecane. 2-methyl-	0	0	0	0	0.0171	0.0022	0.0502	0.002
25.534	Tridecyl methyl ketone	0	0	0.04746	0.0018	0	0	0.0241	0.001
25.536	Heptadecane	0	0	0	0	0.1388	0.0021	0	0
25.55	Octacosane	0.0671	0.0023	0	0	0	0	0	0
25.55	2-Pentadecanone. 6.10.14-trimethyl-	0.0251	0.003	0.04555	0.0035	0	0	0	0
25.555	Heptacosane	0	0	0	0	0.0096	0.001	0	0
25.615	Methyl 7.9-tridecadienyl ether	0	0	0.01093	0.0014	0	0	0.002	3E-04
25.691	2-Tridecanol	0	0	0.0105	0.0017	0	0	0.0037	6E-04
25.821	1.37-Octatriacontadiene	0	0	0.01868	0.002	0	0	0	0
26.505	Tetradecanoic acid	0.0842	0.011	0.07724	0.008	0.0445	0.0041	0.0871	0.007
26.674	2-Hexadecanone	0	0	0	0	0	0	0.0319	0.002
26.721	9-Octadecen-1-ol. (Z)-	0	0	0.00622	0.0002	0	0	0	0
26.822	Acetic acid. chloro-. octadecyl ester	0	0	0.00944	0.0001	0	0	0	0
26.903	Ethyl Oleate	0	0	0.00334	0.0001	0	0	0.009	0.002
26.913	n-Pentadecanol	0	0	0	0	0	0	0.0928	0.009
26.998	Acetic acid. 3.7.11.15-tetramethyl-hexadecyl ester	0.3511	0.031	0.43054	0.042	0.1782	0.018	0.3521	0.028
27.066	Tetradecanoate <ethyl->	0.3321	0.029	0.08972	0.011	0.1575	0.014	0.1544	0.013
27.067	Tetradecanoic acid. ethyl ester	0	0	0	0	0	0	0	0
27.156	Eicosane	0.0128	0.002	0.08013	0.0073	0.0113	0.0025	0.0791	0.008
27.178	2-methyltetracosane	0	0	0.02301	0.0024	0	0	0	0
27.218	Tetracosane <n->	0.0386	0.0027	0	0	0	0	0.012	0.002
27.331	Hexadecane. 2.6.10.14-tetramethyl-	0	0	0.02037	0.0018	0	0	0.0055	1E-04
27.484	Hexadecanal	1.1986	0.12	0.2505	0.026	0.6421	0.057	0.2106	0.024
27.805	Neophytadiene	0	0	0.01446	0.0023	0	0	0.0143	0.003

27.923	4-Chlorobutyric acid. pentadecyl ester	0	0	0	0	0	0	0.0382	0.003
27.924	2-Hexadecene. 3.7.11.15-tetramethyl-. [R-[R*.R*-(E)]]-	0.111	0.012	0.16001	0.013	0.0089	0.0005	0.1187	0.011
28.485	n-Nonadecanol-1	0.0406	0.0031	0	0	0.0443	0.0033	0	0
28.569	Tetradecanoic acid. propyl ester	0.0479	0.0034	0.02283	0.0018	0.0295	0.0029	0.0032	4E-04
29.113	Hexadecanoate <methyl->	0	0	0.03039	0.0022	0	0	0	0
29.216	7.9-Di-tert-butyl-1-oxaspiro(4.5)deca-6.9-diene-2.8-dione	0.0079	0.001	0	0	0	0	0	0
29.603	Hexadecanoic acid <n->	0	0	0.08529	0.0019	0.0082	0.0001	0.0196	0.004
29.619	l-(+)-Ascorbic acid 2.6-dihexadecanoate	0	0	0	0	0.0128	0.0029	0	0
30.071	Palmitate <ethyl->	0.1112	0.009	0.04184	0.0035	0.0629	0.0058	0.0604	0.005
30.155	cis-9-Hexadecenal	0	0	0.02281	0.0018	0	0	0.017	0.003
30.17	E.E.Z-1.3.12-Nonadecatriene-5.14-diol	0	0	0	0	0	0	0.0025	6E-04
30.24	7-Hexadecenal. (Z)-	0	0	0	0	0	0	0.0035	2E-04
30.464	Octadecanal	0	0	0.0036	0.0002	0	0	0.0025	3E-04
31.761	Methyl stearate	0	0	0.01204	0.0051	0	0	0	0

CON - calves fed with milk replacer only (Control); MP - calves fed with milk replacer and supplemented with fermented milk permeate. 0— Baseline measurements; 14— after 14 days of feeding. RT – retention time, min.
Data are presented as mean ± SE (n = 10/group).
